# High cell-surface density of HER2 deforms cell membranes

**DOI:** 10.1038/ncomms12742

**Published:** 2016-09-07

**Authors:** Inhee Chung, Mike Reichelt, Lily Shao, Robert W. Akita, Hartmut Koeppen, Linda Rangell, Gabriele Schaefer, Ira Mellman, Mark X. Sliwkowski

**Affiliations:** 1 Molecular Oncology, Genentech, 1 DNA Way, South San Francisco, California 94080, USA; 2 Research Pathology, Genentech, 1 DNA Way, South San Francisco, California 94080, USA; 3 Translational Oncology, Genentech, 1 DNA Way, South San Francisco, California 94080, USA; 4 Cancer Immunology, Genentech, 1 DNA Way, South San Francisco, California 94080, USA

## Abstract

Breast cancers (BC) with HER2 overexpression (referred to as HER2 positive) progress more aggressively than those with normal expression. Targeted therapies against HER2 can successfully delay the progression of HER2-positive BC, but details of how this overexpression drives the disease are not fully understood. Using single-molecule biophysical approaches, we discovered a new effect of HER2 overexpression on disease-relevant cell biological changes in these BC. We found HER2 overexpression causes deformation of the cell membranes, and this in turn disrupts epithelial features by perturbing cell–substrate and cell–cell contacts. This membrane deformation does not require receptor signalling activities, but results from the high levels of HER2 on the cell surface. Our finding suggests that early-stage morphological alterations of HER2-positive BC cells during cancer progression can occur in a physical and signalling-independent manner.

## Abstract (web summary)

HER2 is frequently overexpressed in breast cancer in association with increased metastatic potential. Here, the authors show that HER2 overexpression causes deformation of cell membranes in a signalling-independent manner that contributes to the disease phenotype by disrupting epithelial features.

## Graphical abstract

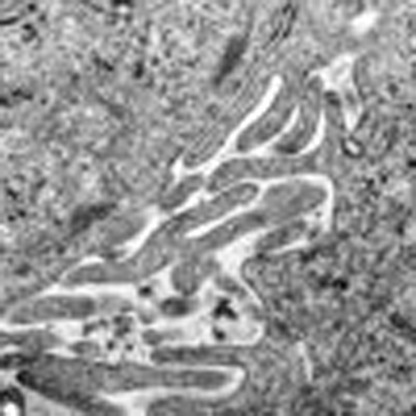

HER2 is a member of the ErbB/HER receptor tyrosine kinase family^1^,^2^,^3^. Gene amplification and overexpression of this protein in breast cancers (BCs) predict poor disease outcome due to elevated metastatic potentials^4^,^5^,^6^,^7^. Studies of the role of HER2 in cancer progression have focused primarily on the signalling activities of HER2. HER2 BC cells with a 3+ immunohistochemistry (IHC) score exhibit high basal levels of receptor kinase activity and phosphorylation, and these induce constitutive activation of the mitogen-activated protein kinase and phosphatidylinositol 3-kinase/Akt pathways^8^. These activities can result in increased cell proliferation^9^,^10^ and invasiveness^11^. Moreover, HER2 3+ BC cells exhibit upregulated epithelial-mesenchymal transition (EMT) inducing transcription factors, such as TWIST and SNAIL, through which cancer cells lose their epithelial characteristics^12^.

These characteristics of HER2 overexpression were acquired from various ensemble experiments, where receptor activities are typically averaged over a very large number of cells. In our previous studies of epidermal growth factor receptor (EGFR) dimerization dynamics at a single-molecule level, we showed that receptor activation by ligand binding is spatially asymmetric on cells overexpressing EGFR (ref. 13). Thus we considered the possibility that individual HER2s may also behave non-uniformly within a single BC cell, and that this heterogeneity might bear a biological significance. Therefore, we employed quantum dot (QD)-based single-receptor tracking and analysis methods in live cells to investigate whether a spatial control is present that may influence the activation of overexpressed HER2s in BC cells. We found that HER2s were distributed in clusters with elongated shapes on cells overexpressing the receptor, while the distribution was more uniform when the expression level was normal. Interestingly, this clustered distribution was independent of HER2 signalling. We found that these patterns resulted from deformed membrane morphologies, which appeared as irregularly shaped ‘finger-like' structures (FLS) in electron micrograph images of HER2 3+ BC cells grown *in vitro* as well as in tissue samples from BC patients. Surprisingly, these finger-like membrane structures were also observed in cells overexpressing signalling-incompetent HER2 mutants, suggesting membrane deformation is induced by the high cell-surface density of HER2 rather than by the receptor's signalling activities. We found that this membrane deformation can reduce the area available for cell contacts with substrates or neighbouring cells. These observations suggest that a non-canonical effect of HER2 overexpression exists that contributes to the disruption of epithelial characteristics exhibited in HER2 3+ BC cells^14^, which is implicated in early-stage cancer progression^15^,^16^.

## Results

### Elongated and clustered HER2 distribution in high expressers

**Figure 1:**
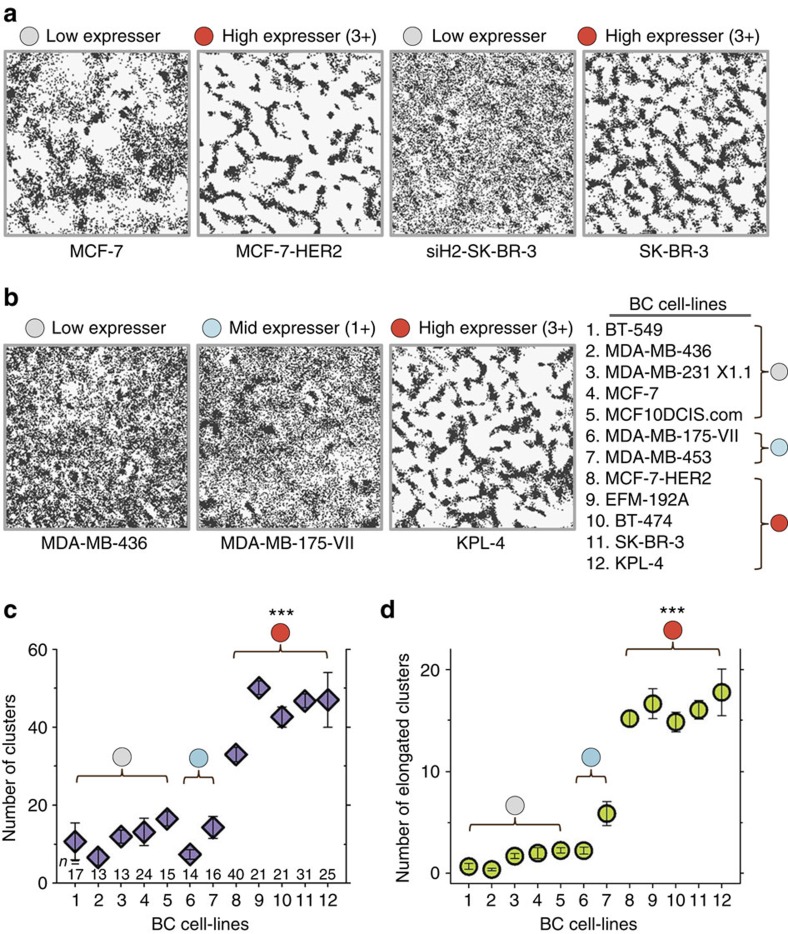
Contrasting HER2 distributions in high versus low expressers. (**a**) A HER2 distribution was constructed from the sum of individual receptor location points (black dots) acquired from tracking single HER2s in the basal cell membrane, for 100 s with an acquisition rate of ∼11 Hz. Each distribution contains ∼60,000 location points in the 8 μm × 8 μm field. It was assumed that the membrane as a whole was stationary during this time window. In high HER2 expressers, MCF-7-HER2 and SK-BR-3 (3+, red circle), HER2 locations appeared ‘clustered' in elongated shapes while they are randomly distributed in the low expressers, MCF-7 and siH2-SK-BR-3 (light grey circle). (**b**) Elongated HER2 clusters are also observed in high expressers (red circle, KPL-4), much less in the mid (light blue circle, MDA-MB-175-VII) and almost invisible in low expressers (light grey circle, MDA-MB-436). All fields are 8 μm by 8 μm. The list of BC cell-lines with different HER2 levels that are used in **c** and **d**). (**c**) The numbers of clusters (mean±s.e.m.) identified in these fields. (**d**) The numbers of elongated clusters (mean±s.e.m.) with the IQ values <0.25. ***P value<10^−6^ by two-sided, unpaired Welch's *t*-test.

We examined the spatial distribution of individual HER2s on live cells that express different levels of HER2. The cells were grouped according to the IHC scoring system, where 0 represents normal level expression, 1+ and 2+ are mid, and 3+ is high expression. A large number of location points of individual HER2s were obtained by tracking single receptors (for ∼ 100 s at a 10.72 Hz acquisition rate) labelled with anti-HER2 Fab:QD (αH2Fab:QD) conjugates using total internal reflection fluorescence microscopy (TIRFM)^13^. We first compared the receptor distribution patterns within the same cell type where HER2 expression levels were artificially altered. Pairs of low and high HER2 expressers were created for two BC cell lines (Fig. 1a). MCF-7 is a low expresser and MCF-7-HER2 is a stable line engineered to overexpress HER2 (3+). SK-BR-3 naturally overexpresses HER2 (3+) and its HER2 level was reduced (to ∼ 1+) by partial small interfering RNA (siRNA) knockdown (siH2-SK-BR-3). To compare the HER2 distribution patterns between cells with widely differing receptor expression levels, the number of receptor locations per image was kept constant (variation <3%) by adjusting the concentration of αH2Fab:QD conjugates. As a result, all images in Fig. 1 contain ∼ 60,000 receptor location points in each 8μm × 8 μm field. Surprisingly, a large fraction of HER2s in the two high expressers appeared ‘clustered' in elongated shapes, whereas the HER2s in their low-expresser counterparts were more uniformly distributed and only a few circular ‘clusters' were found (Fig. 1a). (It should be noted that the term ‘clusters' describes regions on the membrane with high optical densities of HER2, but this does not necessarily correspond with actual receptor clustering.)

We performed similar experiments on other BC cell lines with varying HER2 levels (listed in Fig. 1b) to explore whether this visual ‘clustering' of receptor locations is a general phenomenon in high HER2 expressers. Indeed, the elongated cluster patterns of HER2 were frequently observed in other high expressers (EFM-192A, BT-474 and KPL-4), were less frequent in mid expressers (MDA-MB-175-VII (1+) and MDA-MB-453 (2+)) and rarely observed in low expressers (BT-549, MDA-MB-436, MDA-MB-231X1.1 and MCF10DCIS.com). (HER2 expression levels are compared by WB in Supplementary Fig. 1a). Examples of the HER2 distribution patterns in these cells are shown in Fig. 1b and Supplementary Fig. 1b. We found the receptor level-dependent location patterns are not unique to HER2 as overexpression of EGFR in MCF-7 also generated elongated cluster patterns (Supplementary Fig. 1c).

The elongated HER2 distribution patterns were quantified using a ‘cluster analysis' algorithm that we created. This analysis identifies clustered receptor locations by hierarchically grouping location points that maintain a four-point nearest neighbour connectivity, and enables characterization of the cluster shapes (see Methods section and Supplementary Fig. 2a. for details; very little clustering was found from location points simulated from random walker trajectories). As shown in Fig. 1c, the number of clusters per unit area (8 μm × 8 μm) was significantly greater in high expressers than in mid and low expressers. Since the clusters look more elongated in high expressers (Fig. 1a,b), we characterized the extent of elongation of each cluster by calculating its isoperimetric quotient (IQ), which is the ratio of the area of a given cluster to that of a circle with the same perimeter length. The IQ value for a circle is 1 and decreases as the shape becomes more elongated. As expected, the average IQ was generally smaller for high HER2 expressers than for lower expressers (Supplementary Fig. 2b). Figure 1c shows that high expressers contain significantly more elongated clusters with IQ values smaller than 0.25 compared with those for the low expressers. (This threshold value was determined from the intersection point of the distribution curves of IQ values in high and low HER2 expressers, Supplementary Fig. 2c).

Notably, single-molecule tracking revealed that most HER2s within the elongated clusters were not entirely stationary but could diffuse around within and between the clusters (Supplementary Fig. 2d). This observation suggests that the apparent ‘cluster' of HER2 locations does not indicate an actual cluster (aggregation) of a large number of HER2s; but is instead a result of a confined geometry of the membrane within which these receptors could diffuse.

### Membrane deformation is observed in high HER2 expressers

**Figure 2:**
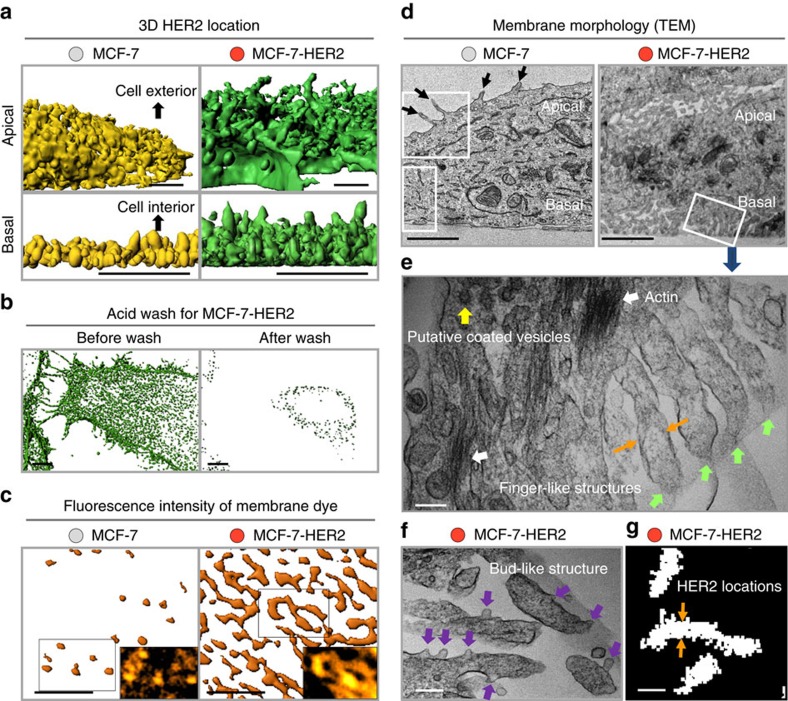
Membrane deformation by high HER2 expression. (**a**) The 3D distributions of QD-labelled HER2s in the apical (top, side view) and basal membranes (bottom, side view) of MCF-7 (yellow) and MCF-7-HER2 (green) created from z-stack snap-shot fluorescence images using confocal microscopy (400 ms bin time). In MCF-7-HER2, HER2s are broadly distributed along the *z* axis in elongated structures on both apical and basal sides, and the 2D projection of these morphologies appears as elongated clusters in the basal membrane as shown in **b** (before urea/acid wash). Scale bar, 3 μm. (**b**) The 2D projection of z-stack images of HER2 locations (green) from the basal membrane to ∼3.3 μm inside of MCF-7-HER2 cells. Most of the αH2Fab:QD conjugates initially bound to HER2s (left) were removed by a 10 min urea/acid wash (right). Scale bar, 3 μm. (**c**) High fluorescence intensity regions of the membranes stained with a lipophilic dye (orange) in live MCF-7 and MCF-7-HER2 cells. Inserts: raw fluorescence images inside the boxes. Scale bar, 4 μm. (**d**) TEM images of different cell membrane morphologies of MCF-7 and MCF-7-HER2. FLS of cell membranes are observed throughout the entire surface of MCF-7-HER2 cells. Zoomed-in images of MCF-7 inside white boxes are shown in Supplementary Fig. 3a. Scale bar, 2 μm. Panel **e** is the zoomed-in (∼ 8 times larger) image of the region in the white box in **d**. Scale bar, 0.2 μm. (**f**) Bud-like membrane protrusions (violet arrows) are observed in MCF-7-HER2. Scale bar, 0.2 μm. (**g**) The width (the distance between two orange arrows) of the elongated clusters of HER2 locations (white dots) is ∼ 0.18 μm (*n*=200), which is similar to that of the FLS in **e**. The precision of determining the centre position^51^ of a single QD is <4 nm, similar to the resolution of the EM. Scale bar, 0.2 μm.

We mapped out the three-dimensional (3D) distributions of HER2 to better characterize their location patterns by confocal microscopy. The 3D distributions of QD-labelled HER2s on live MCF-7 (low expresser) and MCF-7-HER2 (high expresser) cells were created along the optical (*z*) axis for the apical and basal membranes. Side views of these *z*-stack images (Fig. 2a) show irregular and thick HER2 distributions in MCF-7-HER2s (green) in both apical (top panel) and basal (lower panel) membranes. This is in contrast to the overall thin and more planar distributions observed in MCF-7s (yellow) on both surfaces. Interestingly, the 3D shapes of the HER2 locations in MCF-7-HER2s appear to form narrow and elongated structures.

One possible explanation for the thick HER2 distribution along the *z* axis in MCF-7-HER2s is receptor internalization. However, the majority of αH2Fab:QD conjugates attached to HER2s on MCF-7-HER2 cells were removed by a 10 min wash with 2 M urea/50 mM glycine (pH 2.4)^17^ (Fig. 2b). This indicates that most HER2s were membrane bound and exposed to the cell exterior, rather than located inside the cell. In other words, the narrow and elongated 3D receptor location patterns (Fig. 2a) reflect deformed cell membrane morphologies.

If a cell membrane forms an elongated 3D structure, its two-dimensional (2D) optical projection may consist of multiple membrane layers (see the cartoon in Supplementary Fig. 2e). Then, objects associated with the membrane would appear at higher densities in the layered regions, and this could explain the clustered patterns observed in HER2 overexpressers. To test this hypothesis we stained low and high HER2 expressers with a lipophilic dye (1,1′-dioctadecyl-3,3,3′,3′-tetramethylindocarbocyanine perchlorate) that becomes non-specifically incorporated into the membrane. We then compared the fluorescence intensity patterns of this dye in the cell membranes of low and high expressers (Fig. 2c). Numerous higher intensity regions (orange) are observed in the basal membranes of MCF-7-HER2 cells (right), while only a few high intensity regions are found in MCF-7s (left). IQ values of the high intensity membrane regions were smaller (ie, more elongated) in MCF-7-HER2 than in MCF-7 cells (Supplementary Fig. 2f). This was consistent with the patterns of clustered HER2 locations in these cells (Fig. 1a,c), which we also observed from the fluorescence intensity pattern of HER2-GFP when it is overexpressed in MCF-7 cells (Supplementary Fig. 2g). The similar patterns of HER2 and the membrane dye suggest the HER2 clusters likely reflect regions of membrane folding.

We used transmission electron microscopy (TEM) to directly visualize the membrane morphologies of intact MCF-7 and MCF-7-HER2 cells. In MCF-7 cells, the membrane overall is smooth with a few long, irregularly shaped protruded structures (black arrows) on both the apical and basal sides (Fig. 2d, left; zoomed-in images of the areas inside the white rectangles are shown in Supplementary Fig. 3a). In contrast, a larger number of elongated membrane structures, which show association with HER2s (Supplementary Fig. 3b), are observed in MCF-7-HER2 (right), and SK-BR-3 cells (Supplementary Fig. 3c) throughout the entire cell surface. We refer to these protrusions as ‘FLS.' The enlarged image (Fig. 2e) of the area inside the white rectangle in Fig. 2d (right) clearly displays FLS (green arrows), which are very similar to the protrusive cell membrane structures of SK-BR-3 observed by Hommelgaard *et al*.^18^. The FLS were often not significantly associated with actin filaments (Fig. 2e). Besides actin, other actin binding proteins such as villin and ezrin, and phosphatidylinositol (4,5) bis-phosphate (PIP2) that can be associated with these proteins showed little (∼20%) overlap with FLS (immuno-fluorescence (IF) images in Supplementary Fig. 3d–g), suggesting the membrane deformation in these cells was not caused by actin-mediated protrusion in a manner analogous to the brush-border formation^19^. The FLS were also unrelated to caveolin-1 (cav1) mediated membrane tubulation (invagination) that only occurs after cav1 transfection in SK-BR-3 (ref. 20). Interestingly, small bud-like membrane protrusions were frequently observed, which may represent an early-stage in the process that creates FLS (Fig. 2f and Supplementary Fig. 3b). Lateral widths (mean±s.d.; indicated by orange arrows in Fig. 2e,g) of FLS (0.15±0.05 μm) in TEM images of MCF-7-HER2 cells were very similar to those determined for the elongated HER2 clusters (0.18±0.05 μm) obtained from the single-receptor tracking measurements (Fig. 2g).

### Membrane deformation is independent of HER2 signalling

**Figure 3:**
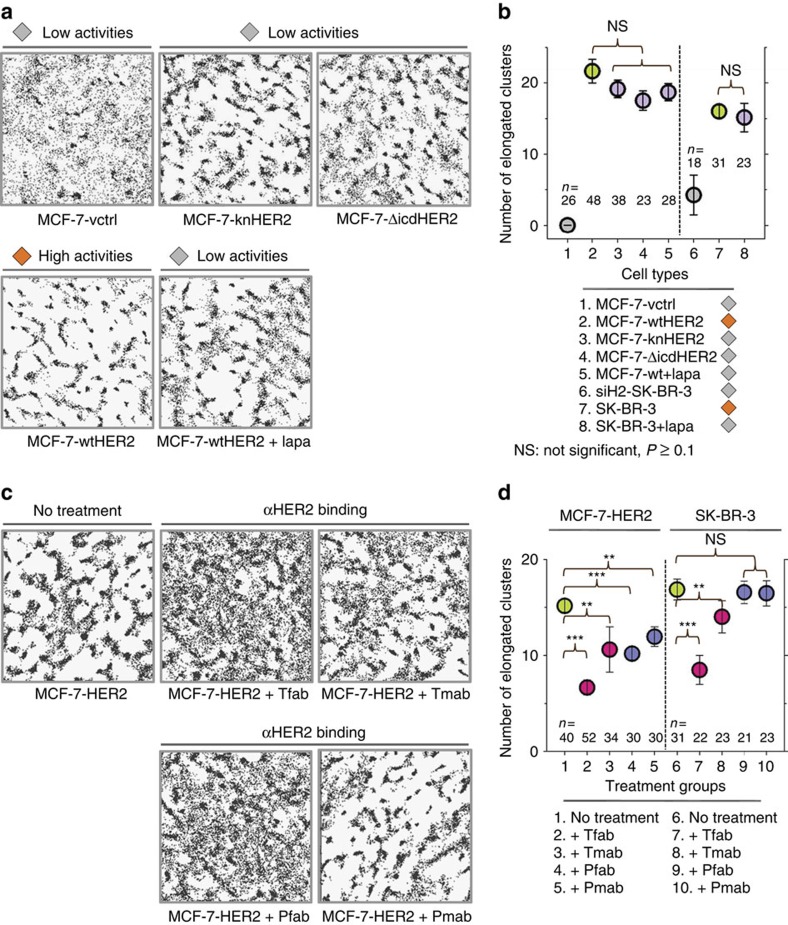
Membrane deformation by HER2 overexpression is independent of HER2 signaling. (**a**) Elongated HER2 clusters are observed in high expressers of signalling-incompetent HER2 mutants, MCF-7-knHER2 and MCF-7-ΔicdHER2, similar to those in wtHER2 high expressers (orange diamond). Clustered HER2 location patterns shown in MCF-7-wtHER2 (orange diamond) were also largely insensitive to 30′ kinase inhibitor (lapatinib) treatment (grey diamond). The occurrence of the elongated HER2 clusters in MCF-7-vctrl (grey diamond) is significantly less. (**b**) Numbers of elongated clusters quantified by cluster analysis in MCF-7 and SK-BR-3 cell variants, indicate that regardless of HER2 signalling activities, 3+ HER2 expression results in significantly greater FLS than low expression (MCF-7-vctrl and siH2-SK-BR-3 cells). Light grey/green/purple circles: low/high/high HER2 levels and low/high/low HER2 signalling activities. (**c**) Effects of 1 day treatments of MCF-7-HER2 cells with trastuzumab fab (Tfab), trastuzumab (Tmab), pertuzumab fab (Pfab) and pertuzumab (Pmab) on elongated cluster patterns of HER2. Tfab and Pfab treatments noticeably down-modulated clustered HER2 patterns in MCF-7-HER2. (**d**) Comparison of the numbers of elongated clusters in MCF-7-HER2 and SK-BR-3 cells untreated/treated with Tfab, Tmab, Pfab and Pmab. Green/magenta/blue circles: untreated/Tfab and Tmab treatments/Pfab and Pmab treatments. ***P value<10^−6^ and **P value<10^−4^ by two-sided, unpaired Welch's *t*-test. All values are mean±s.e.m.

HER2s can become constitutively active when overexpressed. We thus questioned whether the membrane deformation was a consequence of constitutive receptor signalling. To address this possibility, we constructed three different signalling-incompetent mutants of HER2. The three mutations were (i) a point mutation in the kinase domain that disrupts ATP binding (knHER2), (ii) phenylalanine substitutions for seven individual tyrosine auto-phosphorylation sites (7YFHER2), and (iii) deletion of the entire intracellular domain of the receptor (ΔicdHER2). We generated MCF-7 transfectants that stably expressed knHER2 and ΔicdHER2, along with wtHER2 at high levels (3+). We also used all three mutants and wtHER2 for transient expressions in MCF-7 cells. Surprisingly, all MCF-7 cells overexpressing these mutants and wtHER2 exhibited elongated and clustered location patterns of HER2s, while MCF-7-vctrl (transfectants of the control vector) showed random patterns (representative HER2 distributions in the stable and transient transfectants are respectively shown in Fig. 3a and Supplementary Fig. 4a). These results suggest the membrane deformation in high expressers is independent of HER2 signalling. We also examined whether blocking the HER2 kinase activity affects existing FLS by treating the high-expresser MCF-7-wtHER2 with lapatinib, a kinase inhibitor of HER2, for 30 min (93% decrease in HER2 phosphorylation relative to control as assessed by WB, Supplementary Fig. 4b). Despite the reduced HER2 kinase activity, elongated and clustered receptor location patterns in MCF-7-wtHER2 were unaffected overall (Fig. 3a). The membrane deformation in SK-BR-3 was also largely insensitive to reduced HER2 signalling, as elongated cluster patterns of HER2 locations (Supplementary Fig. 4c) as well as FLS in TEM images (Supplementary Fig. 3c), persisted after lapatinib treatment.

Using cluster analysis, we compared the FLS densities in cells expressing 3+ levels of the various signalling-incompetent mutants with those of wtHER2 by calculating the average number of elongated clusters per unit area (8 μm by 8 μm). FLS densities in all 3+ expressers are comparable, and significantly higher than those in their low-expresser counterparts (MCF-7-vctrl and siH2-SK-BR-3). The results in Fig. 3b (for stable transfectants) and Supplementary Fig. 4a (transient transfectants) clearly indicate that FLS formation and maintenance depend on the presence of high HER2 expression in the cell membrane rather than receptor signalling activities.

### HER2 antibodies can affect membrane deformation

Although the membrane deformation is not sensitive to inhibition of HER2 kinase activity, we questioned whether the binding of therapeutic HER2 antibodies could affect the existing FLS in high HER2 expressers. Specifically, we used trastuzumab (Tmab) and pertuzumab (Pmab) that respectively bind to domain IV (ref. 21) and II (ref. 22) of the HER2 ECD (extracellular domain). Additionally, we examined the effects of their fab fragments (Tfab and Pfab), to separate the consequences of the binding itself from other events that could result from antibody bivalency, such as receptor internalization^23^. HER2 distributions in the high expressers (MCF-7-HER2, SK-BR-3, BT-474, and KPL-4) were examined after 2 h and 1 day incubations with Tfab, Tmab, Pfab, and Pmab. Representative HER2 distributions from 1 day treatments in comparison to the untreated groups are shown in Fig. 3c for MCF-7-HER2 cells and in Supplementary Fig. 5a–d for all investigated cells. The distributions for the 2 h time points were similar (data not shown). In all high expressers, FLS densities were generally down-modulated by these four treatments. However, the relative effects of individual treatments varied among different high expressers. In Fig. 3c, HER2 distributions in MCF-7-HER2 after 1 day incubations with Tfab or Pfab appear more random and diffusive compared with those treated with Tmab or Pmab. On the other hand, in SK-BR-3 (Supplementary Fig. 5b,e), while Tfab clearly reduced FLS densities, the effect of Pfab was less obvious. Among the four high expressers, KPL-4 was least responsive to these treatments. FLS densities were obtained from cluster analysis and these are plotted in Fig. 3d for MCF-7-HER2 and SK-BR-3 (1 day treatments) and (Supplementary Fig. 5e) for all investigated cells (both 2 h and 1 day treatments). The fact that HER2 ECD binders can affect FLS at all, and also differently across various cell types is another indication that the membrane deformation results from physical interactions among HER2s, and also between the receptors and other features of the cell membrane, such as other membrane associated proteins or lipid microdomains. We suspected that a steric pressure^24^ generated from the ECDs of diffusing HER2s could be responsible, and tested this idea by overexpressing an ECD deletion mutant of HER2. Interestingly this ECD deletion mutant also induced membrane deformation (Supplementary Fig. 6a–c), suggesting the mechanism is more complex than an ECD crowding effect.

### Membrane deformation appears in HER2 3+ human tumours

**Figure 4:**
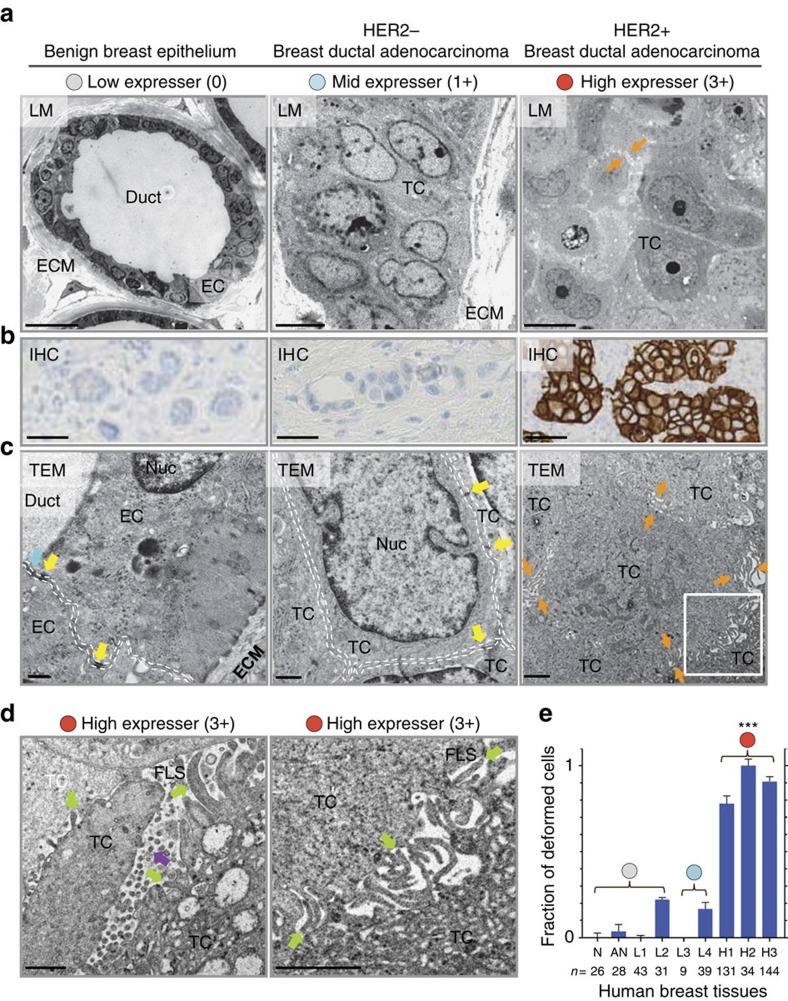
Membrane deformation occurs in HER2 3+ human breast tumours. (**a**–**c**) Human benign breast epithelium (left), HER2 1+ (middle) and HER2 3+ breast ductal adenocarcinoma (right) are shown in (**a**) T-blue stained light microscopy (LM; Scale bars, 20 μm (left) and 5 μm (middle and right)), (**b**) HER2 IHC (scale bars, 40 μm) and (**c**) TEM images (scale bars, 0.5 μm (left and middle) and 2 μm (right)). The TEM images show representative cell morphologies. Tight junctions (cyan arrow), desmosomes (yellow arrows), well-defined cell interfaces (between two white dashed lines), and separation of cells (between two orange arrows) are identified. (**d**) TEM images of two different areas in the HER2 3+ sample. The image on the right is the magnified image of the area in the white rectangle in **c**. Some of the FLS and circular structures are indicated by green and violet arrows, respectively. Scale bars, 1 μm. (**e**) Fractions of deformed cells (mean±s.e.m.) were assessed in a blinded manner. The specimens were from one normal (N), one adjacent normal (AN), four HER2 1+ (L1-L4) and three HER2 3+ (H1-H3) human tissue samples. The fraction was significantly higher in 3+ samples. ***P value<10^−6^ by two-sided, unpaired Welch's *t*-test. EC, epithelial cell; ECM, extracellular matrix; Nuc, nucleus; TC, tumour cell.

We next examined whether the extensive membrane deformation induced by HER2 overexpression observed *in vitro* also occurs in HER2 3+ human breast tumour cells. We examined cell membrane morphologies in breast tissue samples taken from 7 BC patients and one underwent reduction mammoplasty. These samples consisted of invasive ductal adenocarcinoma with HER2 overexpression (3+) and ductal adenocarcinoma with slightly elevated HER2 expression (1+), benign breast epithelia (0) adjacent to a 3+ tumour region and normal breast epithelia (0). Figure 4a shows representative morphological profiles (bright-field microscopy images) of a benign breast epithelium (left), and HER2 1+ (middle) and 3+ (right) breast tumour samples. Both 1+ and 3+ tumour cells show cellular and nuclear pleomorphisms with enlarged nuclei and prominent nucleoli. Also shown are HER2 IHC images that indicate relative HER2 expression levels (Fig. 4b), and high-magnification TEM images (Fig. 4c) from each group. The adjacent benign breast epithelium (left) shows smooth cell membranes that exhibit apical-basolateral cell polarity^25^,^26^ with ducts, and tight junctions (cyan arrow) and desmosomes (yellow arrows) between cells. Interestingly tumour cells with just 1+ expression also show smooth membranes along with well-maintained cell–cell interfaces (white dashed lines; middle), while cell polarity is not apparent. In 3+ tumour cells, cell membranes are considerably deformed (right), epithelial polarity is not observed, and each cell is widely separated from neighbouring cells (orange arrows). Two magnified images (Fig. 4d) show deformed membrane morphologies in greater detail, including irregularly shaped and sized FLS (green arrows), and circular structures (violet arrow), as observed similarly *in vitro*. To establish statistics for HER2 expression-dependent membrane deformation phenotypes in human BC tissues, we counted the cells bearing both deformed membranes and perturbed cell–cell interfaces in 3–5 different regions of each tissue sample in a blinded manner (for details, see Supplementary Fig. 7a–d). The fractions of deformed cells (Fig. 4e) are significantly higher (>80%) in all three HER2 3+ breast tumour tissues than in four 1+ tumour samples (< ∼20%) and adjacent normal (< ∼4%) and normal breast tissues (none). These data clearly demonstrate that the membrane deformation is predominantly associated with HER2 3+ human breast tumour cells.

### Membrane deformation reduces the cell contact area

The fact that most of the HER2 3+ cells with deformed membranes in the TEM images were in loose contact with each other suggests that these signalling-independent alterations of membrane morphology by high HER2 cell-surface densities may play a role in disease progression. Specifically, we speculated that the membrane deformation may reduce the surface area available to cells for interaction with either their surrounding substrates or neighbouring cells. In the former case, the membrane deformation would cause the area available for the formation of focal adhesion (FA) complexes^27^ to decrease. In the latter scenario the contact area eligible for forming cell–cell junctions would be reduced.

**Figure 5:**
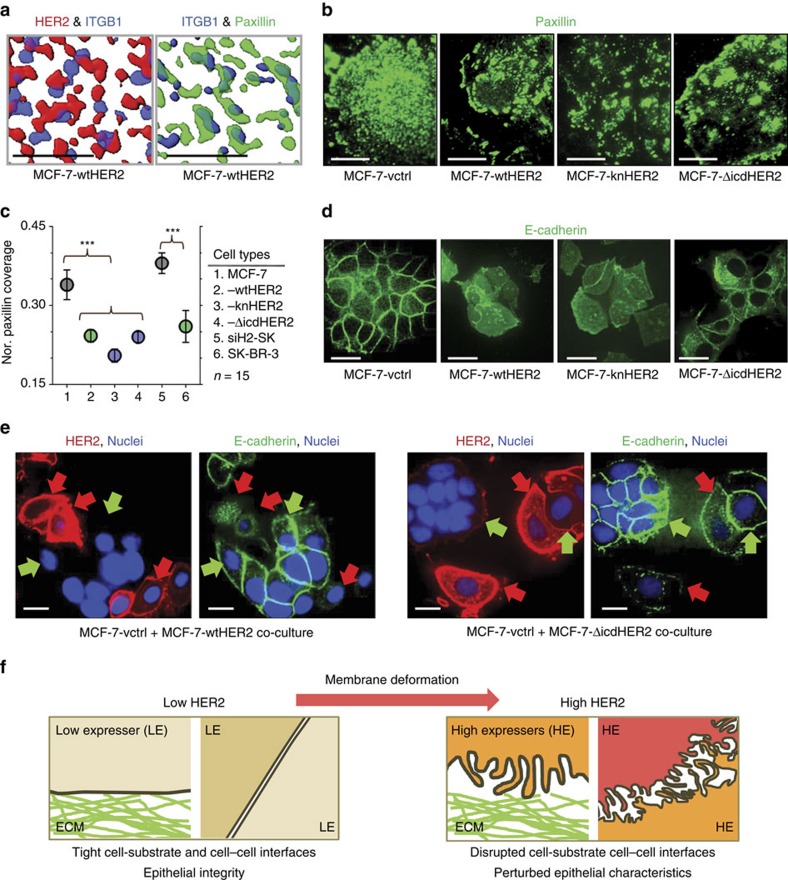
The membrane deformation disrupts cell interfaces. (**a**) Processed images of raw IF data (similar images are shown in Supplementary Fig. 7a,b) acquired from TIRFM imaging of HER2 (red, αH2Fab:QD), integrin beta 1 (ITGB1; blue, αCD29:alexa568) and paxillin (green, αpaxillin:Alexa488) in MCF-7-HER2 cells showing the relative locations of these proteins. Less overlap between HER2 and ITGB1 is observed than between ITGB1 and paxillin. Scale bars, 5 μm. (**b**) IF images of paxillin distributions in basal cell membranes of various MCF-7 transfectants. The Paxillin distributions in all high HER2 expressers (3+; wtHER2 and the two signalling-incompetent HER2 mutants) are irregular compared to the more uniform distribution in the low HER2 expresser MCF-7-vctrl. Scale bars, 20 μm. (**c**) Average cell-surface coverage (mean±s.e.m.) of paxillin clusters (normalized (Nor.). Paxillin coverage) was calculated from the ratio of the area covered with paxillin (FLS greater than ∼13% of the maximum intensity) to the total cell-surface area. ***P value<10^−6^ by two-sided, unpaired Welch's *t*-test. (**d**) Confocal IF images of E-cadherin (green, αE-cadherin:alexa488) in various MCF-7 transfectants. Scale bars, 30 μm. (**e**) Confocal IF images of HER2 (red, αH2Fab:QD) and E-cadherin in MCF-7-wtHER2 (left panels) and MCF-7-ΔicdHER2 (right panels) co-cultured with MCF-7-vctrl to contrast the delocalized patterns of E-cadherin in high HER2 expressers (red arrows) with the localized E-cadherin distributions in low expressers (green arrows). Scale bars, 20 μm. (**f**) A cartoon illustrating how the HER2-induced membrane deformation perturbs cell–substrate and cell–cell interfaces, and thus disrupts epithelial integrity. Shades of beige and red represent different cells.

To address the reduced surface area with surrounding substrates, the distributions of FA complexes were compared with those of FLS by IF. We used integrin β1 (ITGB1) and paxillin to mark FA complexes, which are, respectively, an initiator of the complex assembly and an adaptor that can directly associate with the integrin^28^. Indeed the IF images (Supplementary Fig. 8a) show little overlap between FA complexes and the elongated regions of high HER2 fluorescence signal (FLS) in MCF-7-wtHER2 cells. As expected, ∼70% of the high-density regions of ITGB1 (blue) co-localize with paxillin (green; also shown in Supplementary Fig. 8b), while neither ITGB1 nor paxillin significantly overlaps with the high HER2 regions (red) in Fig. 5a. The reduced localizations were not due to a decrease in protein expression levels (Supplementary Fig. 8c). These results suggest the membrane deformation would reduce the cell-surface coverage of FA complexes.

To further validate the relationship between HER2 overexpression driven membrane deformation and reduced cell–substrate contact areas, we quantified how HER2 expression levels and receptor signalling activities in the stable cell lines affect the cell-surface coverage of paxillin. IF images for paxillin in MCF-7 stable transfectants are shown in Fig. 5b. In low expressers (MCF-7-vctrl and siH2-SK-BR-3), small paxillin clusters (<0.5 μm^2^) (ref. 29) densely covered the entire basal cell surfaces. However, in all high expressers of wtHER2 (MCF-7-wtHER2 and SK-BR-3) and the signalling-incompetent mutants (MCF-7-knHER2, and MCF-7-ΔicdHER2), as well as lapatinib treated SK-BR-3, the cell-surface densities of small paxillin clusters in the central regions were significantly reduced compared with low expressers. (See Supplementary Fig. 8d for IF images for paxillin in SK-BR-3 variants and Supplementary Fig. 8e for the comparison of the size-distributions of paxillin clusters between high and low HER2 expressers.) Similar results were obtained when MCF-7 cells transiently overexpressed wtHER2, knHER2, 7YFHER2 and ΔicdHER2 (see IF images of paxillin staining in these cells in Supplementary Fig. 8f). The ratios of the areas covered with paxillin relative to the total basal cell-surface area across the various cell types are compared in Fig. 5c. These results were independent of the substrates on which the cells were grown. We found the cell-surface coverage of paxillin was also reduced in high-expresser MCF-7-wtHER2 cells compared with MCF-7-ctrl cells, when these cells were grown on collagen (Supplementary Fig. 8g). The correlation between the reduced paxillin coverage and high HER2 expression, regardless of the receptor's signalling activities, suggests that this reduction resulted from the HER2 overexpression induced membrane deformation.

Because FLS were observed on the entire cell surface of high HER2 expressers by TEM (Fig. 2d, and Supplementary Figs 3c and 6d), it is likely they may also perturb the formation of cell–cell contacts. To examine this, we visualized the locations of E-cadherin and ZO-1, which are present in adherens and tight junctions, respectively. In MCF-7 (low expresser), E-cadherin (green, Fig. 5d for stable transfectants) and ZO-1 (along with E-cadherin as shown in Supplementary Fig. 9a for transient transfectants) are clearly localized at the cell interfaces. This indicates the presence of solid cell–cell contacts. In contrast, for all high HER2 (red) expresser counterparts, regardless of receptor signalling capability, these localizations are greatly reduced (Fig. 5d and Supplementary Fig. 9a). Again this reduction was not caused by a decrease in the protein level (Supplementary Fig. 8c). The disparate localization patterns of E-cadherin are clearly shown in the IF images of high expressers (wtHER2 and ΔicdHER2) co-cultured with MCF-vctrl on glass cover-slips (Fig. 5e) and in basement membrane extract matrices (Supplementary Fig. 9b; also see the relative fluorescence intensity profiles of HER2 vs E-cadherin cell-surface localizations). In these images, the adhesion protein is significantly more delocalized in the high expressers than in the low expressers. Importantly the reduced localizations were not caused by the loss of E-cadherin nor did they result in up-regulation of mesenchymal markers such as vimentin (see WB data in Supplementary Fig. 8c).

We performed a similar series of experiments on MCF10DCIS.com (DCIS.com), a cell line originally derived from the MCF10A line, which forms predominantly comedo-type ductal carcinoma *in situ* (DCIS) *in vivo*^30^,^31^. DCIS is a non-invasive early-stage BC, but DCIS lesions with HER2 overexpression are likely to rapidly progress to invasive BC (ref. 14). As we expected, HER2 overexpression in DCIS.com cells also reduced the cell-surface coverages of paxillin and E-cadherin (see IF data in Supplementary Fig. 9c). In these experiments, we consistently saw an inverse relationship between high HER2 surface expression and membrane localization of E-cadherin. All these data clearly demonstrate that cell interfaces can be perturbed by physical deformation of their membranes without transcriptional alterations that downregulate adhesion proteins^32^,^33^,^34^,^35^. Additionally, the membrane deformation induced epithelial disruption are consistent with the morphological alterations of HER2 +3 BCs observed in DCIS (refs 36, 37).

## Discussion

Most human BCs originate from epithelial cells and progress through multiple histological stages to become invasive and eventually spread to distant organs. This progression involves disruption of normal epithelial morphology and increased dissemination potential of tumour cells as a result of various genetic alterations^16^,^38^,^39^,^40^. HER2 overexpression in BCs is known to facilitate this progression^4^,^5^,^6^,^7^. Specifically HER2 overexpression is associated with development of histological alterations of early-stage BC tissues^36^,^37^, and the overexpression in DCIS (early-stage cancer) lesions predicts invasive disease progression^14^.

In this work, we describe a previously uncharacterized effect of HER2 overexpression that leads to disruption of epithelial integrity by causing a morphological deformation of cell membranes into FLS (summarized in Fig. 5f). These effects did not downregulate E-cadherin, which is implicated in invasive cancer progression by virtue of its ability to induce EMT (refs 12, 32, 33, 34, 35). Cell interface disruptions are known early-stage events in the EMT induced by HER2 overexpression in BC (ref. 12). In addition to the previously characterized effects of HER2 signalling on EMT, our data suggest that these interface disruptions may also result from signalling-independent, physical alterations of cell membranes by HER2 overexpression. This event is independent of, and orthogonal to, other well-described consequences of HER2 signalling activities^4^,^6^,^7^,^8^,^34^. The membrane deformation we observed is not unique to HER2 overexpression as we found similar structures in cells overexpressing EGFR. We hypothesize that this type of membrane alteration might represent an additional mechanism through which tumour cells overexpressing these receptor tyrosine kinases become less attached to their surroundings and more prone to acquiring an invasive phenotype.

Other types of membrane deformation have been associated with the pathogenesis of cancer. For example, invadopodia are actin-rich membrane structures that assist the invasiveness of tumour cells by recruiting matrix proteases to the tips of the protrusive structures and degrading the ECM (refs 41, 42). Another membrane protrusion generated by cytoskeleton-regulatory proteins is called the filopodia-like protrusion, which is distinguished from filopodia by the ubiquitous distribution of ITGB1 and the long persistence of the protrusion. The integrin-rich filopodia-like protrusion becomes an assembly site for mature adhesions, and the establishment of adhesions in turn causes FAK-dependent mitogen-activated protein kinase/ERK (extracellular signal-regulated kinases) activation. This contributes to metastatic colonization of cancer cells in the post-extravasation stage^43^. In contrast to these actin-mediated membrane protrusions, the FLS formations we observed were not dependent on the level of HER2 signalling nor were they strictly associated with actin. These observations suggest a different functional significance for this membrane morphological alteration.

We hypothesize FLS generation is mediated by physical processes, possibly involving elevated concentration of HER2 in lipid microdomains^44^,^45^, which then leads to membrane bending^46^,^47^,^48^,^49^,^50^ through protein–protein and protein–lipid interactions. This possibility is the subject of future investigation.

## Methods

### Materials

The αHER2 and αgD antibodies were mouse IgG1 clones of 7C2.B9 (0.333 mg ml^−1^ of the fab fragments was used for conjugation with QDs), and 5B6 (12.0 mg ml^−1^; 1:200 dilution was used for WB) from Genentech. Other antibodies were purchased, including αpY1248HER2 (rabbit, GeneTex, GTX25654; 1:500 dilution for WB), αbeta-tubulin (mouse, Sigma, T9026; 1:10,000 dilution for WB), αpaxillin (mouse; ThermoFisher (TF), AHO0492; 1:200 dilution for WB and IF), αbeta-Actin:Alexa488 conjugates (mouse, Sigma, R37110; 2 drops/ml for IF), αVimentin (rabbit; Cell Signaling Technology (CST), 5741S; 1:1,000 dilution for WB), αE-Cadherin (mouse; Life Technologies, 33–4,000; 1:500 dilution for WB), αE-Cadherinalexa488 (rat; eBioscience, 53-3249-82; 5 μg ml^−1^ for IF), and αCD29 (mouse; eBioScience, 14-0291-82; 5 μg ml^−1^ for IF) and DAPI (4′,6-Diamidino-2-Phenylindole, Dihydrochloride, Biotium, 40043; 1:200 dilution for IF). HER2 siRNA oligos (SignalSilence HER2/ErbB2 siRNAs II, CST, 6282; 20 nM), lipophilic dye (DiI (1,1′-dioctadecyl-3,3,3′,3′-tetramethylindocarbocyanine perchlorate, TF, D282; 2 μM), c-terminus HER2 antibody (rabbit, Dako, A0485; 1:2,000 dilution for WB), HER2-GFP (Origene, RG212583), αVillin (rabbit; Abcam, ab52102; 1:50 dilution for IF), αEzrin:Alexa488 (Rabbit, Abcam, ab198520; 1:100 dilution for IF ), αPIP2 (Mouse, Echelon, Z-P045; 1:100 dilution for IF) and basement membrane extract extracellular matrices (Reduced Growth Factor Basement Membrane Extract, Type 2; Cultrex, 3533-010-02) were purchased and used as the protocols suggested.

### Mutagenesis and transfection

For transient transfection, wtHER2 (full length HER2) as well as knHER2 (K753M), 7YFHER2 (Y to F substitutions at Y877, Y1023, Y1139, Y1195, Y1221, Y1222 and Y1248), ΔicdHER2 (HER2 intracellular domain truncation: 1-748aa), and ΔecdHER2 (HER2 extracellular domain truncation: 639-1255aa) genes that were fused with N-terminal gD epitope tags were cloned into pRK5 expression vectors after site-directed mutagenesis. Primers used for each constructs are following.

knHER2: Forward: 5′- CCAGTGGCCATCATGGTGTTGAGGGAA -3′; 7YFHER2: Forward: 5′- CATTGACGAGACAGAGTTCCATGCAGATGGGGGCAAG -3′; Reverse: 5′- CTGCTGGGGTACCAGAAACTCCTCAGCATCCAC -3′; Forward: 5′- GCCCCCAGCCTGAATTTGTGAACCAGCCAGATG -3′; Reverse: 5′- CTCCTCCCTGGGGTGTCAAAAACTCGGGGTTCTCCACGGC-3′; Forward: 5′- CAGCCCAGCCTTCGACAACCTCTTTTTCTGGGACCAGGACCCACCAGAGCG -3′; Reverse: 5′- CTGGCACGTCCAGACCCAGAAACTCTGGGTTCTCTGCCG -3′; ΔicdHER2: Forward: 5′- AGACTGCTGCAGTGAATTCAGCTGGTGGAG -3′; ΔecdHER2: Forward: 5′- GTCCTGGACCAGCTGCTCGAGAAGGGCTGCCCCGCCGAGCAGAGA -3′; Reverse: 5′- GCCAAGCTTCTGCAGGTCGACGGTATCGATTGAATTCCCCCCTTC -3′;

gD is derived from the HSV (herpes simplex virus) envelope glycoprotein D. MCF-7, SK-BR-3 and MCF10DCIS.com cells were transfected using FuGENE 6 Transfection Reagent (Promega), with the DNA constructs or siRNA oligos mixed in Opti-MEM (Life Technologies). Cells grown in 2D were incubated in the transfection mixture containing normal growth medium, RPMI (Roswell Park Memorial Institute) 1640 (MCF-7) and DMEM (SK-BR-3) with 10% foetal bovine serum (FBS) and 1% L-Glutamine, and DMEM:F12 50:50 (MCF10DCIS.com) with 5% horse serum and 1% L-Glutamine for ∼2 or 3–4 days, for the constructs or siRNA oligos, respectively. For generation of stable cell lines, wtHER2, knHER2 and ΔicdHER2 were subcloned into the PLPCX retroviral vector (BD Biosciences). The retroviral constructs were transfected into the Phoenix amphotropic packaging cell line using Lipofectamine Plus reagent according to the recommendation of the manufacturer (Invitrogen). Viral particles were harvested 48 h after transfection and MCF7 and MCF10 DCIS.com cells were infected. Cell pools expressing HER2 or HER2 mutants were selected using puromycin (Clontech Laboratories, 0.75 μg ml^−1^ for MCF-7 and 1.5 μg ml^−1^ for DCIS.com transfectants).

### Cell-line culture

Cell-lines used in this work were purchased from ATCC and DSMZ, and were maintained and authenticated using Short Tandem Repeat analysis by the Genentech central cell repository (gCell), which ensures that the cells were free of contamination. MCF-7, MCF-7-HER2, MDA-MB-231 X1.1, MDA-MB-453, EFM-192A and KPL-4 cells and maintained in RPMI 1640 with 10% FBS, and 1% L-Glutamine in 5% CO_2_ at 37 °C. MDA-MB-436, MDA-MB-175 VII and SK-BR-3 were kept in DMEM with 10% FBS, and 1% L-Glutamine. For MCF10DCIS.com culture, DMEM:F12, with 5% horse serum, and 1% L-Glutamine was used. The condition was kept constant during the whole-imaging experiments and throughout cell preparation procedures for WB and IF. For imaging, cells were plated on glass-bottom dishes (MatTek; glass thickness: No. 1.5).

### Fab:QD conjugation

Fab fragments of the HER2 antibody (7C2.B9 clone) were conjugated to QDs according to the protocol of the Qdot Antibody Conjugation Kit (Life Technologies), with minor modifications. 1.75 μl of a freshly dissolved 20 mM solution of sulfo-SMCC (Thermo Scientific) in DMSO was added to 62.5 μl (0.5 nmoles) of an 8 μM stock solution of amino-PEG-QD605 (Life Technologies). The mixture was incubated at room temperature (RT) for 1 h. In parallel, 100 μg of Fabs was diluted in 300 μl PBS and reduced by adding 6.1 μl of 20 mM dithiothreitol/water solution at RT for 30 min. The sulfo-SMCC derivatized QDs were separated from excess unreacted sulfo-SMCC by passing the solutions over NAP-5 desalting columns (GE Healthcare) pre-equilibrated in 50 mM HEPES, pH 7.2, 150 mM NaCl. Similarly, the reduced Fab fragments were passed over NAP-5 columns pre-equilibrated in the same HEPES/NaCl buffer to remove dithiothreitol. The derivatized QDs and reduced Fab fragments were mixed and allowed to react at RT for 2 h. Remaining free maleimide groups were deactivated by 2-mercaptoethanol (final concentration of 100 μM) at RT for 30 min. The Fab:QD conjugates were then concentrated by ultrafiltration (Pierce Protein Concentrators, Thermo Scientific), and separated from unconjugated Fab fragments by gel filtration (Superdex G200, GE Healthcare). The final conjugate pool in PBS was brought to 50% glycerol and kept at 4 °C. The Fab:QD ratio of a representative QD conjugation reaction was determined using ^125^I-labelled Fab. The Fab concentration in the conjugates was measured from radioactive counts and the QD concentration was determined by the absorbance of QD605 at 600 nm. The Fab:QD ratio by this method was 1:1.3.

### Western blots

Cells were lysed in SDS sample buffer supplemented with reducing reagent (Life Technologies), separated on 4–12% SDS-PAGE gels (Life Technologies) and transferred to nitrocellulose membranes (Bio-Rad). Membranes were blocked in TBS containing 0.2% Tween 20 and 2% BSA, and then probed with primary antibodies. After washing, the membranes were incubated with HRP conjugated secondary antibodies (Life Technologies), and the blots were visualized using a chemiluminescent substrate (GE Healthcare).

### Optical imaging and image processing methods

For single-molecule tracking and IF imaging for ITGB1 and paxillin, the TIRFM was performed using a Nikon Eclipse TE2000 inverted microscope with a × 100/1.49NA Plan Apo objective (Nikon). Samples were illuminated by 430, 488, and 561 nm lines of solid-state lasers (Andor Technology) and images were acquired by the iXon back-illuminated EMCCD camera (Andor Technology). The OptoSplit II Image Splitter (Cairn research) was used to simultaneously record images with two different spectral windows. For confocal imaging for 3D HER2 location patterns, 3D spheroid assays and IF imaging for E-cadherin and ZO-1, we used a Yokogawa CSU-X1 Spinning Disk Unit (Andor Technology) with an iXon DU-897-BV monochrome CCD (Andor Technology). The TIRFM and the spinning disk confocal units were installed at the ports on the opposite sides of the inverted microscope to allow for switching between two different image modes during visualization of the same cells. The imaging environment was maintained at 5% CO_2_, 37 °C. Tracking of individual molecules and cells and image rendering were performed using Imaris (Bitplane) or Image-J (NIH). The cluster analysis was created in Matlab (Mathworks).

### Calculation of isoperimetric quotient

We quantified the elongation of deformed membrane structures by assessing the IQ of each clustered HER2 location, which correlates with the elongation. IQ is the ratio of the area *A* of the region to the area of a circle having the same perimeter *L*, 4*πA*/*L*^2^. When the shape is a circle, the area is maximized with a given length of the perimeter and therefore IQ is 1. The only parameters to measure for this analysis are the perimeter and the area of an object, and therefore this is a very simple method to assess how an object shape can deviate from a circle.

### Cluster analysis

HER2 distributions (individual HER2 locations) were obtained from ∼100 s-long trajectories of single QD-labelled HER2s on the basal membrane of living cells collected at 10.72 Hz using TIRFM imaging. The density of labelled HER2 locations was chosen to be ∼60,000 per the area of 8 μm by 8 μm so that there are a sufficient number of location points to determine clusters. Each location point inside the boundary of a cluster has at least four neighbour location points within a radius (*δ*) of 0.025 μm. A region after a relay of these groupings (hierarchical grouping) of location points was assigned as a cluster if 55–65 % of the area inside the boundary of this region is filled with circles with the radius of *δ*/2, depending on a cell (the overlapping areas are all ignored; this process is graphically illustrated in Supplementary Fig. 2a). After Gaussian filtering to smooth the boundary, clusters with their area smaller than 0.25 μm^2^ were discarded. Then the number of the clusters (Fig. 1c), the area, and the perimeter of individual clusters were measured. In addition, using the area and perimeter values, the shape of the clusters was assessed by calculating IQ. Simulated location points of 100 random walkers for 600 time points showed random distributions (Supplementary Fig. 2a).

### Immuno-fluorescence

Cells were maintained in their normal growth media at 37 °C before treatment with Fixation and Permeabilization reagent (Life Technologies). Cells were incubated with primary antibodies for 3 h. For HER2 and ITGB1, primary antibodies that recognize the ECDs of these receptors were added to live cells before fixation. Bound antibodies were labelled with fluorescent secondary antibodies (Alexa 488/568 conjugated, Life Technologies), and imaged with TIRFM or confocal microscopy.

### Transmission electron microscopy

Human specimens were acquired from commercial sources (the MT group). Informed consent and Institutional Review Board (IRB) approval were obtained based on a representation and warranty from the vendors. For cell-line experiments, cells were grown in 6-well plates, washed in PBS and then fixed in 1/2 Karnovsky's fixative (2% paraformaldehyde, 2.5% glutaraldehyde in 0.1 M sodium cacodylate buffer, pH 7.2). For simultaneous visualization of HER2, αHER2 (7C2.B9; mouse) and αmouse secondary antibody conjugated with 12 nm gold particles (Jackson ImmunoResearch Laboratories Inc.) were used. The samples were then post-fixed in 1% aqueous osmium tetroxide, stained with 1% uranyl acetate and dehydrated through a series of ethanol steps (50, 70, 90, 95 and 100%) followed by propylene oxide. During the propylene oxide step, cell sheets were harvested from the 6-well dish and transferred to a 1.5 ml eppendorf tube for embedding in Eponate 12 (Ted Pella). The samples were cured at 65 °C. Semi-thin (500 nm) and ultra-thin (80 nm) sections were obtained with an Ultracut microtome (Leica). The semi-thin sections were stained with Toluidine Blue and examined by bright-field microscopy (magnifications: 1000x) to obtain an overview of the area examined by TEM. Ultra-thin sections were counter stained with 0.2% lead citrate and examined in a JEOL JEM-1400 TEM at 120 kV. Digital images were captured with a GATAN Ultrascan 1000 CCD camera.

### Data availability

The data that support the findings of this study are available upon request from the corresponding authors (I.C. and M.X.S.).

## Additional information

**How to cite this article:** Chung, I. *et al*. High cell-surface density of HER2 deforms cell membranes. *Nat. Commun.* 7:12742 doi: 10.1038/ncomms12742 (2016).

## Supplementary Material

Supplementary Information Supplementary Figures 1-9

## References

[b1] Coussens L. . Tyrosine kinase receptor with extensive homology to egf receptor shares chromosomal location with neu oncogene. Science 230, 1132–1139 (1985). 2999974 10.1126/science.2999974

[b2] Slamon D. J. . Studies of the her-2/neu proto-oncogene in human-breast and ovarian-cancer. Science 244, 707–712 (1989). 2470152 10.1126/science.2470152

[b3] Yarden Y. & Sliwkowski M. X. Untangling the ErbB signalling network. Nat. Rev. Mol. Cell Biol. 2, 127–137 (2001). 11252954 10.1038/35052073

[b4] Hynes N. E. ErbB2 activation and signal transduction in normal and malignant mammary cells. J. Mammary Gland Biol. Neoplasia 1, 199–206 (1996). 10887493 10.1007/BF02013643

[b5] Olayioye M. A. Update on HER-2 as a target for cancer therapy - intracellular signaling pathways of ErbB2/HER-2 and family members. Breast Cancer Res. 3, 385–389 (2001). 11737890 10.1186/bcr327 PMC138705

[b6] Yarden Y. Biology of HER2 and its importance in breast cancer. Oncology 61, 1–13 (2001). 11694782 10.1159/000055396

[b7] Yu D. H. & Hung M. C. Overexpression of ErbB2 in cancer and ErbB2-targeting strategies. Oncogene 19, 6115–6121 (2000). 11156524 10.1038/sj.onc.1203972

[b8] Moasser M. M. The oncogene HER2: its signaling and transforming functions and its role in human cancer pathogenesis. Oncogene 26, 6469–6487 (2007). 17471238 10.1038/sj.onc.1210477 PMC3021475

[b9] Neve R. M. . Effects of oncogenic ErbB2 on G1 cell cycle regulators in breast tumour cells. Oncogene 19, 1647–1656 (2000). 10763821 10.1038/sj.onc.1203470

[b10] Lenferink A. E., Busse D., Flanagan W. M., Yakes F. M. & Arteaga C. L. ErbB2/neu kinase modulates cellular p27(Kip1) and cyclin D1 through multiple signaling pathways. Cancer Res. 61, 6583–6591 (2001). 11522658

[b11] Johnson E. . HER2/ErbB2-induced breast cancer cell migration and invasion require p120 catenin activation of Rac1 and Cdc42. J. Biol. Chem. 285, 29491–29501 (2010). 20595387 10.1074/jbc.M110.136770 PMC2937981

[b12] Giordano A. . Epithelial-mesenchymal transition and stem cell markers in patients with HER2-positive metastatic breast cancer. Mol. Cancer Ther. 11, 2526–2534 (2012). 22973057 10.1158/1535-7163.MCT-12-0460 PMC3500676

[b13] Chung I. . Spatial control of EGF receptor activation by reversible dimerization on living cells. Nature 464, 783–787 (2010). 20208517 10.1038/nature08827

[b14] Roses R. E. . HER-2/neu Overexpression as a predictor for the transition from *in situ* to invasive breast cancer. Cancer Epidemiol. Biomarkers 18, 1386–1389 (2009). 10.1158/1055-9965.EPI-08-1101 PMC2713817 19383888

[b15] Thiery J. P. Epithelial-mesenchymal transitions in cancer onset and progression. Bull. Acad. Natl Med. 193, 1969–1978 (2009). 20666011

[b16] Valastyan S. & Weinberg R. A. Tumor metastasis: molecular insights and evolving paradigms. Cell 147, 275–292 (2011). 22000009 10.1016/j.cell.2011.09.024 PMC3261217

[b17] Austin C. D. . Endocytosis and sorting of ErbB2 and the site of action of cancer therapeutics trastuzumab and geldanamycin. Mol. Biol. Cell 15, 5268–5282 (2004). 15385631 10.1091/mbc.E04-07-0591 PMC532009

[b18] Hommelgaard A. M., Lerdrup M. & van Deurs B. Association with membrane protrusions makes ErbB2 an internalization-resistant receptor. Mol. Biol. Cell 15, 1557–1567 (2004). 14742716 10.1091/mbc.E03-08-0596 PMC379255

[b19] Tyska M. J. . Myosin-1a is critical for normal brush border structure and composition. Mol. Biol. Cell 16, 2443–2457 (2005). 15758024 10.1091/mbc.E04-12-1116 PMC1087248

[b20] Verma P., Ostermeyer-Fay A. G. & Brown D. A. Caveolin-1 induces formation of membrane tubules that sense actomyosin tension and are inhibited by polymerase I and transcript release factor/cavin-1. Mol. Biol. Cell 21, 2226–2240 (2010). 20427576 10.1091/mbc.E09-05-0417 PMC2893987

[b21] Cho H. S. & Leahy D. J. Structure of the extracellular region of HER3 reveals an interdomain tether. Science 297, 1330–1333 (2002). 12154198 10.1126/science.1074611

[b22] Franklin M. C. . Insights into ErbB signaling from the structure of the ErbB2-pertuzumab complex. Cancer Cell 5, 317–328 (2004). 15093539 10.1016/s1535-6108(04)00083-2

[b23] Rudnick S. I. & Adams G. P. Affinity and avidity in antibody-based tumor targeting. Cancer Biother. Radiopharm. 24, 155–161 (2009). 19409036 10.1089/cbr.2009.0627 PMC2902227

[b24] Stachowiak J. C. . Membrane bending by protein-protein crowding. Nat. Cell Biol. 14, 944–949 (2012). 22902598 10.1038/ncb2561

[b25] Bissell M. J. & Bilder D. Polarity determination in breast tissue: desmosomal adhesion, myoepithelial cells, and laminin 1. Breast Cancer Res. 5, 117–119 (2003). 12631393 10.1186/bcr579 PMC154157

[b26] Debnath J. & Brugge J. S. Modelling glandular epithelial cancers in three-dimensional cultures. Nat. Rev. Cancer 5, 675–688 (2005). 16148884 10.1038/nrc1695

[b27] Burridge K., Fath K., Kelly T., Nuckolls G. & Turner C. Focal adhesions - transmembrane junctions between the extracellular-matrix and the cytoskeleton. Annu. Rev. Cell Biol. 4, 487–525 (1988). 3058164 10.1146/annurev.cb.04.110188.002415

[b28] Schaller M. D. Paxillin: a focal adhesion-associated adaptor protein. Oncogene 20, 6459–6472 (2001). 11607845 10.1038/sj.onc.1204786

[b29] Gardel M. L., Schneider I. C., Aratyn-Schaus Y. & Waterman C. M. Mechanical integration of actin and adhesion dynamics in cell migration. Annu. Rev. Cell Dev. Biol. 26, 315–333 (2010). 19575647 10.1146/annurev.cellbio.011209.122036 PMC4437624

[b30] Dawson P. J., Wolman S. R., Tait L., Heppner G. H. & Miller F. R. MCF10AT: a model for the evolution of cancer from proliferative breast disease. Am. J. Pathol. 148, 313–319 (1996). 8546221 PMC1861604

[b31] Debnath J., Muthuswamy S. K. & Brugge J. S. Morphogenesis and oncogenesis of MCF-10A mammary epithelial acini grown in three-dimensional basement membrane cultures. Methods 30, 256–268 (2003). 12798140 10.1016/s1046-2023(03)00032-x

[b32] Onder T. T. . Loss of E-cadherin promotes metastasis via multiple downstream transcriptional pathways. Cancer Res. 68, 3645–3654 (2008). 18483246 10.1158/0008-5472.CAN-07-2938

[b33] Thiery J. P., Acloque H., Huang R. Y. J. & Nieto M. A. Epithelial-mesenchymal transitions in development and disease. Cell 139, 871–890 (2009). 19945376 10.1016/j.cell.2009.11.007

[b34] Hanahan D. & Weinberg R. A. Hallmarks of cancer: the next generation. Cell 144, 646–674 (2011). 21376230 10.1016/j.cell.2011.02.013

[b35] Lamouille S., Xu J. & Derynck R. Molecular mechanisms of epithelial-mesenchymal transition. Nat. Rev. Mol. Cell Biol. 15, 178–196 (2014). 24556840 10.1038/nrm3758 PMC4240281

[b36] van de Vijver M. J. . Neu-protein overexpression in breast cancer. Association with comedo-type ductal carcinoma *in situ* and limited prognostic value in stage II breast cancer. N. Engl. J. Med. 319, 1239–1245 (1988). 2903446 10.1056/NEJM198811103191902

[b37] Allred D. C. . Overexpression of HER-2/neu and its relationship with other prognostic factors change during the progression of *in situ* to invasive breast cancer. Hum. Pathol. 23, 974–979 (1992). 1355464 10.1016/0046-8177(92)90257-4

[b38] Elenbaas B. . Human breast cancer cells generated by oncogenic transformation of primary mammary epithelial cells. Gene Dev. 15, 50–65 (2001). 11156605 10.1101/gad.828901 PMC312602

[b39] Shapiro I. M. . An EMT-driven alternative splicing program occurs in human breast cancer and modulates cellular phenotype. PLoS Genet. 7, e1002218 (2011). 21876675 10.1371/journal.pgen.1002218 PMC3158048

[b40] Thiery J. P. & Lim C. T. Tumor dissemination: an EMT affair. Cancer Cell 23, 272–273 (2013). 23518345 10.1016/j.ccr.2013.03.004

[b41] Weaver A. M. Invadopodia: specialized cell structures for cancer invasion. Clin. Exp. Metastas 23, 97–105 (2006). 10.1007/s10585-006-9014-1 16830222

[b42] Paz H., Pathak N. & Yang J. Invading one step at a time: the role of invadopodia in tumor metastasis. Oncogene 33, 193–202 (2013). 10.1038/onc.2013.393 PMC3969876 24077283

[b43] Shibue T., Brooks M. W., Inan M. F., Reinhardt F. & Weinberg R. A. The outgrowth of micrometastases is enabled by the formation of filopodium-like protrusions. Cancer Discov. 2, 706–721 (2012). 22609699 10.1158/2159-8290.CD-11-0239 PMC3418422

[b44] Nagy P. . Lipid rafts and the local density of ErbB proteins influence the biological role of homo- and heteroassociations of ErbB2. J. Cell Sci. 115, 4251–4262 (2002). 12376557 10.1242/jcs.00118

[b45] Sezgin E. . Partitioning, diffusion, and ligand binding of raft lipid analogs in model and cellular plasma membranes. Biochim. Biophys. Acta 1818, 1777–1784 (2012). 22450237 10.1016/j.bbamem.2012.03.007

[b46] Farsad K. & De Camilli P. Mechanisms of membrane deformation. Curr. Opin. Cell Biol. 15, 372–381 (2003). 12892776 10.1016/s0955-0674(03)00073-5

[b47] McMahon H. T. & Gallop J. L. Membrane curvature and mechanisms of dynamic cell membrane remodelling. Nature 438, 590–596 (2005). 16319878 10.1038/nature04396

[b48] Zimmerberg J. & Kozlov M. M. How proteins produce cellular membrane curvature. Nat. Rev. Mol. Cell Biol. 7, 9–19 (2006). 16365634 10.1038/nrm1784

[b49] Rim J. E., Ursell T. S., Phillips R. & Klug W. S. Morphological phase diagram for lipid membrane domains with entropic tension. Phys. Rev. Lett. 106, 057801 (2011). 21405437 10.1103/PhysRevLett.106.057801 PMC3178462

[b50] Bassereau P., Phillips R. & Schwille P. Focus on the physics of the cell membrane. New J. Phys. 14, 055021 (2012).

[b51] Lew M. D. & Moerner W. E. Azimuthal polarization filtering for accurate, precise, and robust single-molecule localization microscopy. Nano Lett. 14, 6407–6413 (2014). 25272093 10.1021/nl502914k PMC4245985

