# Supplementary material for: High cell-surface density of HER2 deforms cell membranes
Source: Nat Commun. 2016 Sep 7;7:12742. doi: 10.1038/ncomms12742 (PMC5023959; doi:10.1038/ncomms12742)
Supplement: Supplementary Information — Supplementary Figures 1-9 [file ncomms12742-s1.pdf]

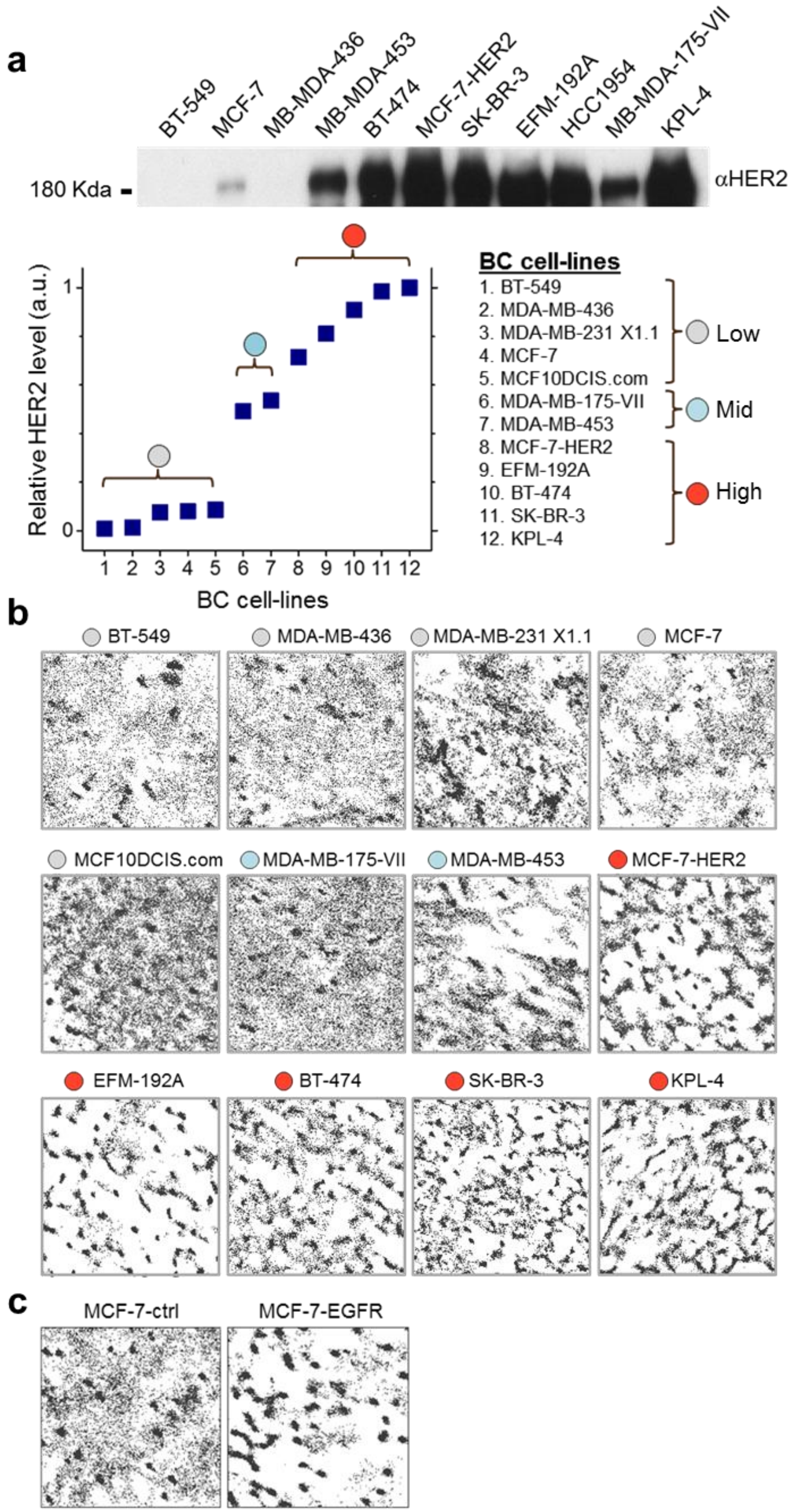
a
BT-549
MCF-7
MB-MDA-436
MB-MDA-453
BT-474
MCF-7-HER2
SK-BR-3
EFM-192A
HCC1954
MB-MDA-175-VII
KPL-4
180 Kda
αHER2
Relative HER2 level (a.u.)
BC cell-lines
BC cell-lines
1. BT-549
2. MDA-MB-436
3. MDA-MB-231 X1.1
4. MCF-7
5. MCF10DCIS.com
6. MDA-MB-175-VII
7. MDA-MB-453
8. MCF-7-HER2
9. EFM-192A
10. BT-474
11. SK-BR-3
12. KPL-4
Low
Mid
High
b
BT-549
MDA-MB-436
MDA-MB-231 X1.1
MCF-7
MCF10DCIS.com
MDA-MB-175-VII
MDA-MB-453
MCF-7-HER2
EFM-192A
BT-474
SK-BR-3
KPL-4
c
MCF-7-ctrl
MCF-7-EGFR

Supplementary Figure 1. HER2 distribution patterns in low, mid, and high HER2 expressing BC cell lines.

(a) Comparison of HER2 expression levels in various breast cancer cell lines. Western Blot (top) of total HER2 levels in various BC cell-lines, normalized by cell number (samples equivalent to 50,000 cells were loaded in each lane). HER2 expression levels were assessed by densitometry (bottom). In addition, for MDA-MB-231 X1.1 and MCF10DCIS.com cells FACS was used to measure HER2 expression relative to MCF-7 and KPL-4 cells. Relative HER2 expression levels across cell lines were plotted (bottom). Light gray, light blue, and red circles indicate low, mid, and high HER2 expressers, respectively. (b) HER2 distribution patterns in low, mid, and high HER2 expressing BC cell lines. Each image contains ~ 60,000 location points of QD-labeled single HER2s (black dots) in areas of 8 μm × 8 μm. These location points were obtained from trajectories collected at 10.72 Hz for ~ 100 s in the basal membranes. Note that similar numbers of HER2s were tracked, and therefore these images do not reflect differences in total HER2 expression. HER2s are fairly uniformly distributed with relatively few clustered patterns in the low and mid expressers (light gray and light blue circles). In the high expressers (red circles), a large fraction of HER2s appear clustered in elongated shapes. (c) Overexpression of EGFR in MCF-7 also results in more clustered and elongated location patterns of this receptor than in MCF-7 cells transfected with control vectors.

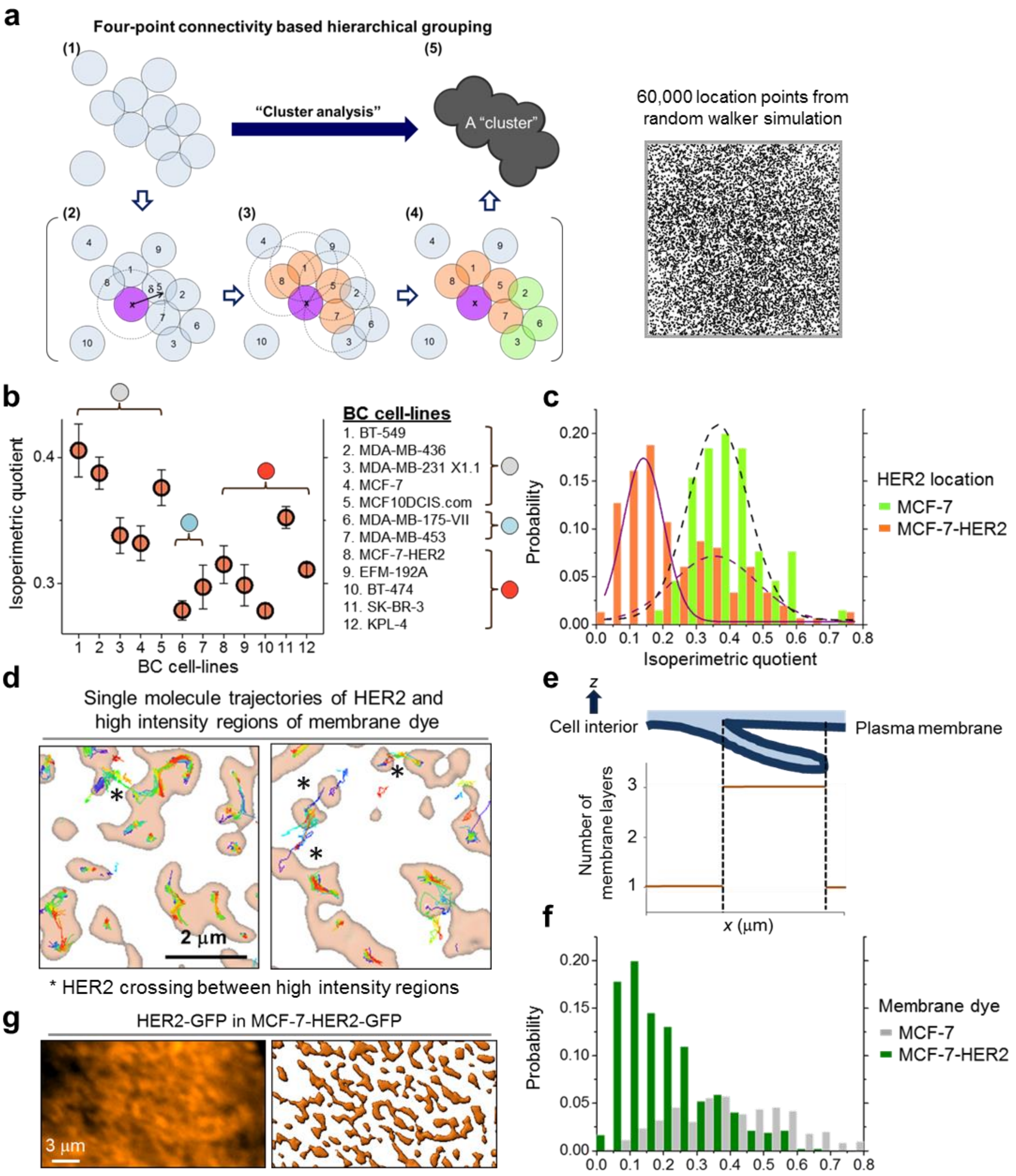
a
Four-point connectivity based hierarchical grouping
(1)
(5)
"Cluster analysis"
A "cluster"
(2)
(3)
(4)
60,000 location points from random walker simulation
b
Isoperimetric quotient
BC cell-lines
BC cell-lines
1. BT-549
2. MDA-MB-436
3. MDA-MB-231 X1.1
4. MCF-7
5. MCF10DCIS.com
6. MDA-MB-175-VII
7. MDA-MB-453
8. MCF-7-HER2
9. EFM-192A
10. BT-474
11. SK-BR-3
12. KPL-4
c
Probability
Isoperimetric quotient
HER2 location
MCF-7
MCF-7-HER2
d
Single molecule trajectories of HER2 and high intensity regions of membrane dye
2 μm
* HER2 crossing between high intensity regions
e
z
Cell interior
Plasma membrane
Number of membrane layers
x (μm)
f
Probability
Isoperimetric quotient
Membrane dye
MCF-7
MCF-7-HER2
g
HER2-GFP in MCF-7-HER2-GFP
3 μm

Supplementary Figure 2. Determination of a threshold isoperimetric quotient (IQ) value for elongated HER2 clusters.

(a) Left: An illustration of the clustering analysis. 11 location points are represented by light blue circles with the same radius of $\delta/2$ in panel (1). “Clustered” location points are identified by sequentially grouping these circles according to whether they maintain ≥ 4 point connectivity, as illustrated in panels (2-5). In this example, circles neighboring the purple circle in panel (2), whose center points are located within a distance of δ (large dotted circle) from the center point (‘x’) of the purple circle, are those labeled 1, 5, 7, and 8. Since the total number of neighboring circles that satisfy this distance condition is ≥ 4, these circles (in orange in panel (3)) are grouped with the purple circle. The same distance condition (as shown by the large dotted circles in (3)) is then applied to the orange circles (1, 5, 7, and 8) for further grouping. As a result, circles 5 and 7 are shown to have ≥ 4-point connectivity with their neighbors, and these new circles (2, 3, and 6; in green) are then combined with the previous group, as shown in (4). Through this process, a total of 8 circles are grouped as a cluster (the dark gray object in (5)). Right: a representative image of 60,000 location points from 300 time points of 200 random walker movements. The number of elongated clusters measured from these images was 0.042 ± 0.007. This value is about 10 times less than that (0.37 ± 0.11) obtained from MDA-MB-436 cells, which is the cell line that exhibited the least number of elongated clusters among the BC cell lines we investigated. (b) Calculations of the isoperimetric quotients of clusters. To quantify the level of elongation of each HER2 cluster, isoperimetric quotient (IQ) values were calculated for individual clusters in the basal cell membranes of the cell-lines listed (right). The IQ is defined as the ratio of the area of a given cluster to the area of a circle bearing the same perimeter length of the cluster. The IQ value for a circle is 1 and becomes smaller as the shape becomes more elongated. The IQ values (mean

± s.e.m.) generally decrease with increasing HER2 expression. (c) Determination of a threshold IQ value for an elongated shape of a HER2 cluster. The probabilities of HER2 clusters with various isoperimetric quotient (IQ) values for MCF-7 (light green bars) and MCF-7-HER2 (orange bars) were plotted with a bin-size of 0.05. Relative to the distribution for MCF-7, which can be fit with a Gaussian function (center position (error) at: 0.37 (0.01), black dashed curve), a large population of clusters with smaller IQ values was observed for MCF-7-HER2. This biphasic distribution was fit to two Gaussians with center positions (errors) at 0.14 (0.01) for the purple solid curve, and 0.36 (0.06) for the purple dashed curve. A IQ value at 0.25 marked the intersection of the two distributions, and this was used as a threshold IQ to segregate populations of elongated clusters within a distribution. (d) Single molecule trajectories of HER2s followed for ~ 25 s at 10.72 Hz acquisition rate overlapped with the high intensity membrane regions stained by the membrane dye (1,1'-dioctadecyl-3,3,3',3'-tetramethylindocarbocyanine perchlorate) in SK-BR-3 cells that overexpress HER2. HER2s are observed to diffuse within and between (indicated by *) the high intensity regions. (e) A schematic representation of a folded membrane that depicts variations in the number of membrane layers in the z projection. If the receptor distribution is extended into a third dimension due to deformation of the membrane in high HER2 expressers, a 2D projection of this membrane region may contain a local increase in the number of membrane layers. This cartoon shows the side profile (*x*-*z* plane) of an imaginary deformed membrane (the dark blue line represents the plasma membrane bilayer) that forms a finger like structure (FLS). The graph below indicates the changes in the number (orange lines) of membrane layers when projected in *z*. (f) Relative distributions of IQ values for high intensity regions of cell membranes stained by a lipophilic dye between low and high HER2 expressers. Distributions of isoperimetric quotient (IQ) values of high intensity regions of the membrane dye

in MCF-7 (gray bars) and MCF-7-HER2 (green). The bin-size is 0.05 as in (a). Similar to what was observed for clustered patterns of HER2 location points, a large population of the high intensity regions with small IQ values (elongated clusters) are found in MCF-7-HER2 cells. This is consistent with the idea that the elongated cluster patterns of HER2 locations result from membrane deformation. (g) As illustrated in the schematics in Supplementary Fig. 2e, if the membrane is folded, HER2-GFP in the folded regions will be sampled more and as a result, the GFP signal will be higher. The left panel shows the raw image of HER2-GFP transfected in MCF-7 cells, and the right panel displays a processed image that filters the high intensity regions.

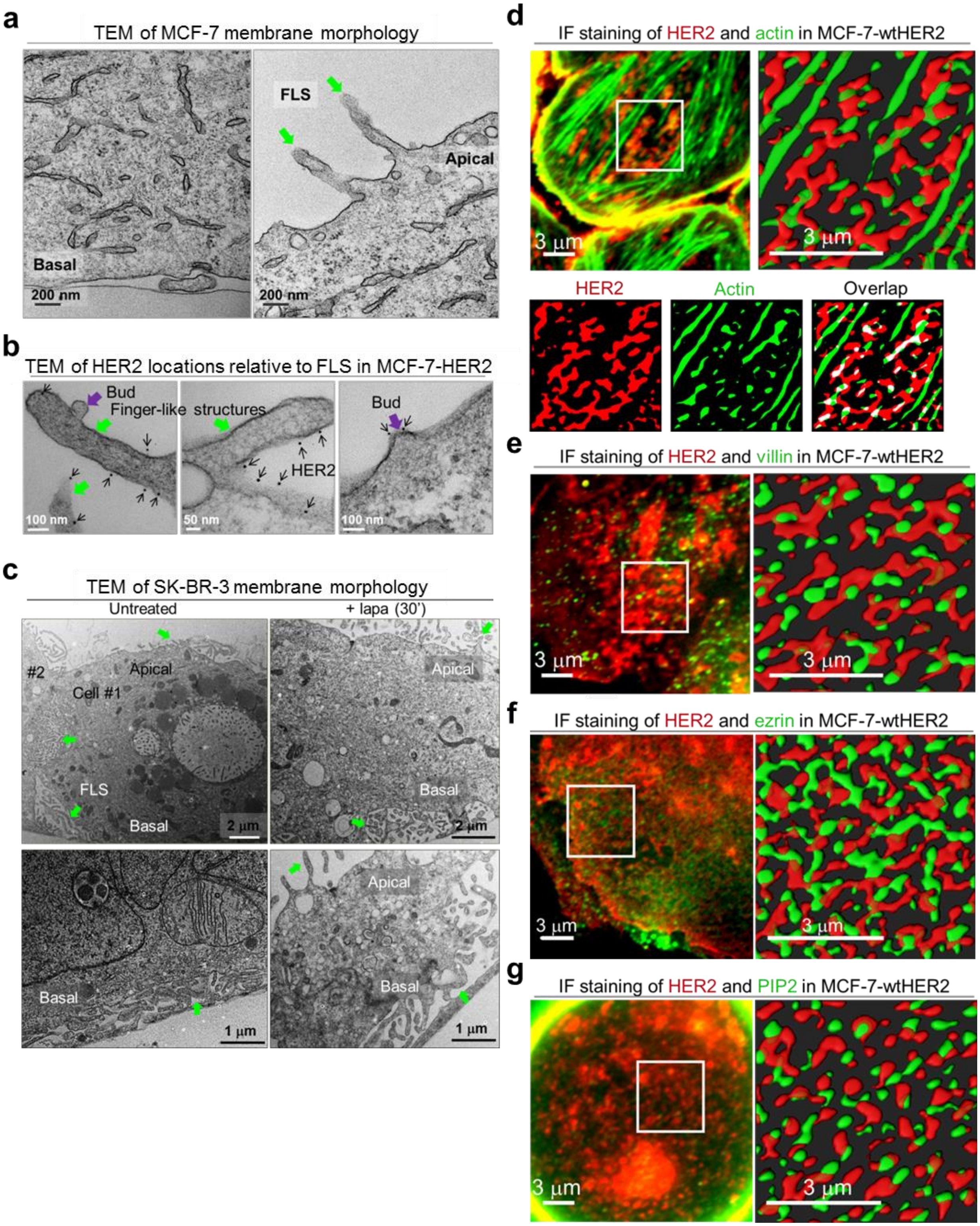

a
TEM of MCF-7 membrane morphology
FLS
Apical
Basal
200 nm
200 nm
b
TEM of HER2 locations relative to FLS in MCF-7-HER2
Bud
Finger-like structures
HER2
Bud
100 nm
50 nm
100 nm
c
TEM of SK-BR-3 membrane morphology
Untreated
+ lapa (30')
#2
Apical
Cell #1
FLS
Basal
2 μm
Apical
Basal
2 μm
Basal
1 μm
Apical
Basal
1 μm
d
IF staining of HER2 and actin in MCF-7-wtHER2
3 μm
3 μm
HER2
Actin
Overlap
e
IF staining of HER2 and villin in MCF-7-wtHER2
3 μm
3 μm
f
IF staining of HER2 and ezrin in MCF-7-wtHER2
3 μm
3 μm
g
IF staining of HER2 and PIP2 in MCF-7-wtHER2
3 μm
3 μm

Supplementary Figure 3. FLS are observed on cells overexpressing HER2.

(a) TEM images of basal (left) and apical (right) membranes of MCF-7 cells. These membranes are relatively smooth, although FLS (indicated by green arrows) occasionally appear in the membranes of these low HER2 expressers. (b) TEM images of HER2 locations relative to FLS in MCF-7-HER2 cells. Gold labeled HER2s (black arrows) associate with FLS (green arrows) and bud-like membrane structures (purple arrows) on MCF-7-HER2 cells. (c) TEM images of membrane morphologies of untreated and lapatinib treated SK-BR-3 cells. TEM images of sections of SK-BR-3 cells that were untreated and treated with lapatinib (a kinase inhibitor of HER2) for 30 min. Similar to what was seen with the membrane morphologies of MCF-7-HER2 cells, FLS (some of them are noted with green arrows) frequently appear along the surfaces (both apical and basal), and between cells as shown in the left images in (a)). Both untreated and treated cells show similar amounts of FLS, suggesting that reducing HER2 signaling did not perturb existing FLS. (d-g) Immuno-fluorescence imaging of actin (d), villin (e), ezrin (f), and PIP2 (g) (all in semi-transparent green), relative to HER2 (αH2:Alexa conjugates; red) in MCF-7-wtHER2 by TIRFM. For colocalization analyses, the raw image (upper left, d) in the white box was rendered to produce a binary image (upper right, d) after smoothing and applying a threshold filter to the intensities (greater than ~ 13% of the maximum values) and sizes (larger than 0.14 μm^2) of each object. The overlapping regions (white, lower right) between the red (HER2; lower left) and green (actin; lower middle) clusters were used to calculate the percentage of HER2 regions colocalizing with actin or other proteins. This scheme was also used for the all IF images for villin, ezrin, and PIP2. We found only ~ 20% of the entire area of red is colocalized with green clusters in the binary images, indicating that for the most part these proteins are not much associated with the FLS in MCF-7-wtHER2 cells.

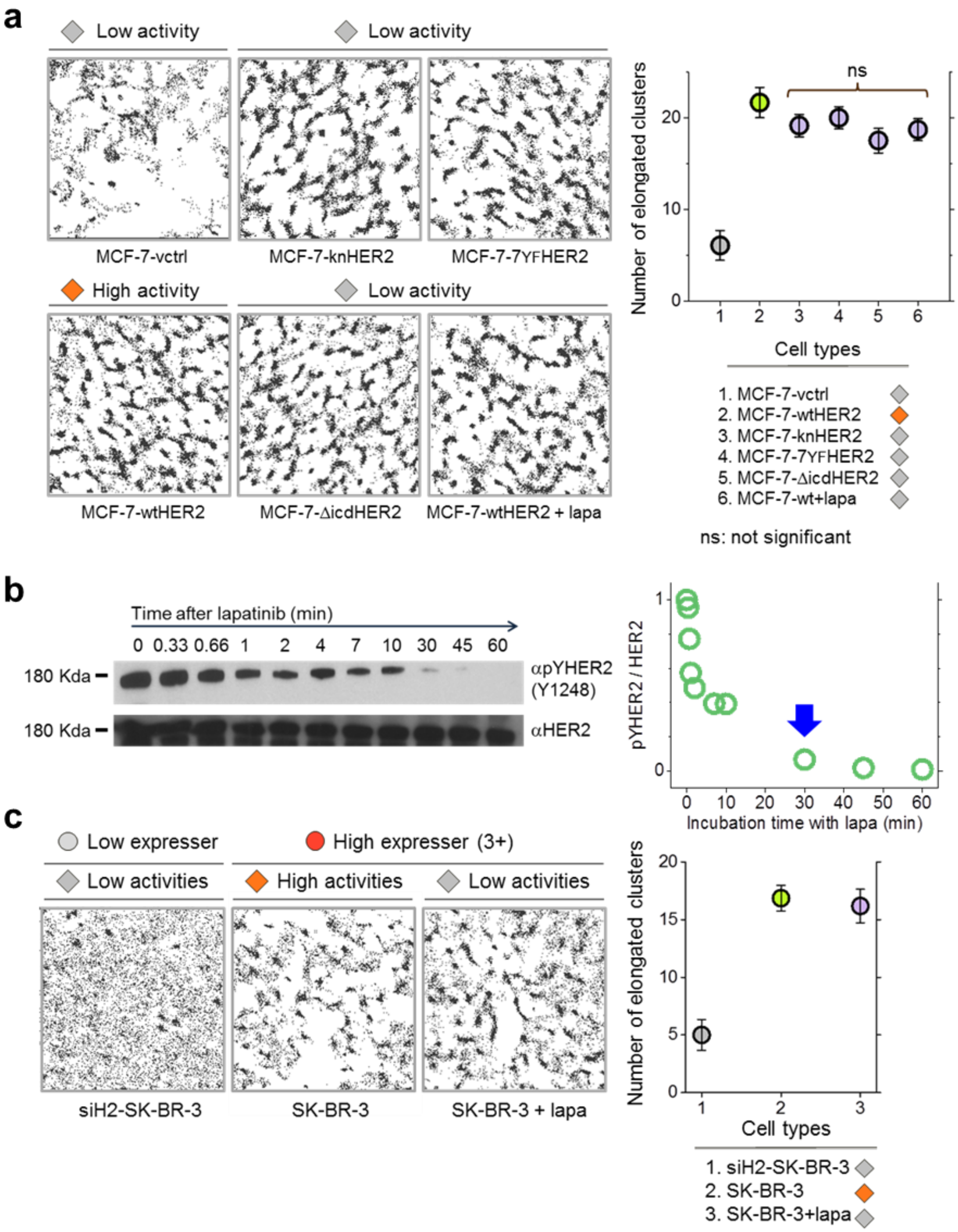

a
Low activity
Low activity
MCF-7-vctrl
MCF-7-knHER2
MCF-7-7YFHER2
High activity
Low activity
MCF-7-wtHER2
MCF-7-ΔicdHER2
MCF-7-wtHER2 + lapa
Number of elongated clusters
ns
Cell types
1. MCF-7-vctrl
2. MCF-7-wtHER2
3. MCF-7-knHER2
4. MCF-7-7YFHER2
5. MCF-7-ΔicdHER2
6. MCF-7-wt+lapa
ns: not significant
b
Time after lapatinib (min)
0 0.33 0.66 1 2 4 7 10 30 45 60
180 Kda
αpYHER2 (Y1248)
180 Kda
αHER2
pYHER2 / HER2
Incubation time with lapa (min)
c
Low expresser
High expresser (3+)
Low activities
High activities
Low activities
siH2-SK-BR-3
SK-BR-3
SK-BR-3 + lapa
Number of elongated clusters
Cell types
1. siH2-SK-BR-3
2. SK-BR-3
3. SK-BR-3+lapa

Supplementary Figure 4. HER2 overexpression induced membrane deformation is independent of HER2 signaling activities.

(a) (Left) Three HER2 mutants, knHER2, 7YFHER2, and ΔicdHER2, along with wtHER2 and an empty vector (vctrl) were transiently overexpressed (3+) in MCF-7 cells. All MCF-7 cells overexpressing these mutants and wtHER2 exhibit similar elongated and clustered location patterns of HER2s. A 30 min lapatinib (kinase inhibitor) treatment did not affect the pattern. Elongated and clustered HER2 patterns are significantly less frequent in MCF-7-vctrl cells. (Right) Plot of the number of FLS per unit area of membrane for the various cell types. The FLS densities ("number of elongated clusters" per unit area) in all 3+ HER2 expressers quantified by cluster analysis are similar and occur with greater frequency than in the control, irrespective of the HER2 signaling activities. These results suggest that FLS formation and maintenance depend on the high HER2 density, rather than on the receptor's kinase activities. Gray circles: low HER2 expression, low constitutive HER2 signaling; green circles: high HER2 expression, high constitutive signaling; purple circle: high HER2 expression, low constitutive signaling. (b) (Left) Time-dependent changes of the pYHER2 level after lapatinib treatment. MCF-7-HER2 cells treated with 10 μM lapatinib (final concentration) for varying amounts of time were run on WB and probed with a phospho-specific HER2 antibody (pY1248). Cells were maintained in full growth medium before and after treatment. (Right) HER2 pY1248 levels were normalized to total HER2 (yellow circles) to compensate for loading errors. At ~ 30 min the net receptor phosphorylation level is reduced to ~ 7% of basal levels. At this time, single HER2 tracking was performed for ~ 100 s to assess the effect of reduced HER2 phosphorylation on the FLS. (c) FLS formation requires high HER2 expression, but it is insensitive to lowered HER2 signaling activities. The three images (8 μm by 8 μm) show the HER2 distributions in SK-BR-3 variants.

The left image is from a low HER2 expresser (gray circle) generated by partial HER2 knockdown (siH2-SK-BR-3). The middle image is an untreated high expresser (red circle), and the right image is a high expresser (red circle) pre-treated for 30 min with lapatinib. The untreated high expresser (middle) contains constitutive HER2 signaling activities (orange diamond), while the low expresser and lapatinib treated SK-BR-3 do not (gray diamonds). Cluster analysis was employed to quantify the number of elongated clusters that indicate FLS (right graph), and these data show FLS are insensitive to the 30 min lapatinib treatment in SK-BR-3, just as what was observed in MCF-7-wtHER2 cells in a similar experiment.

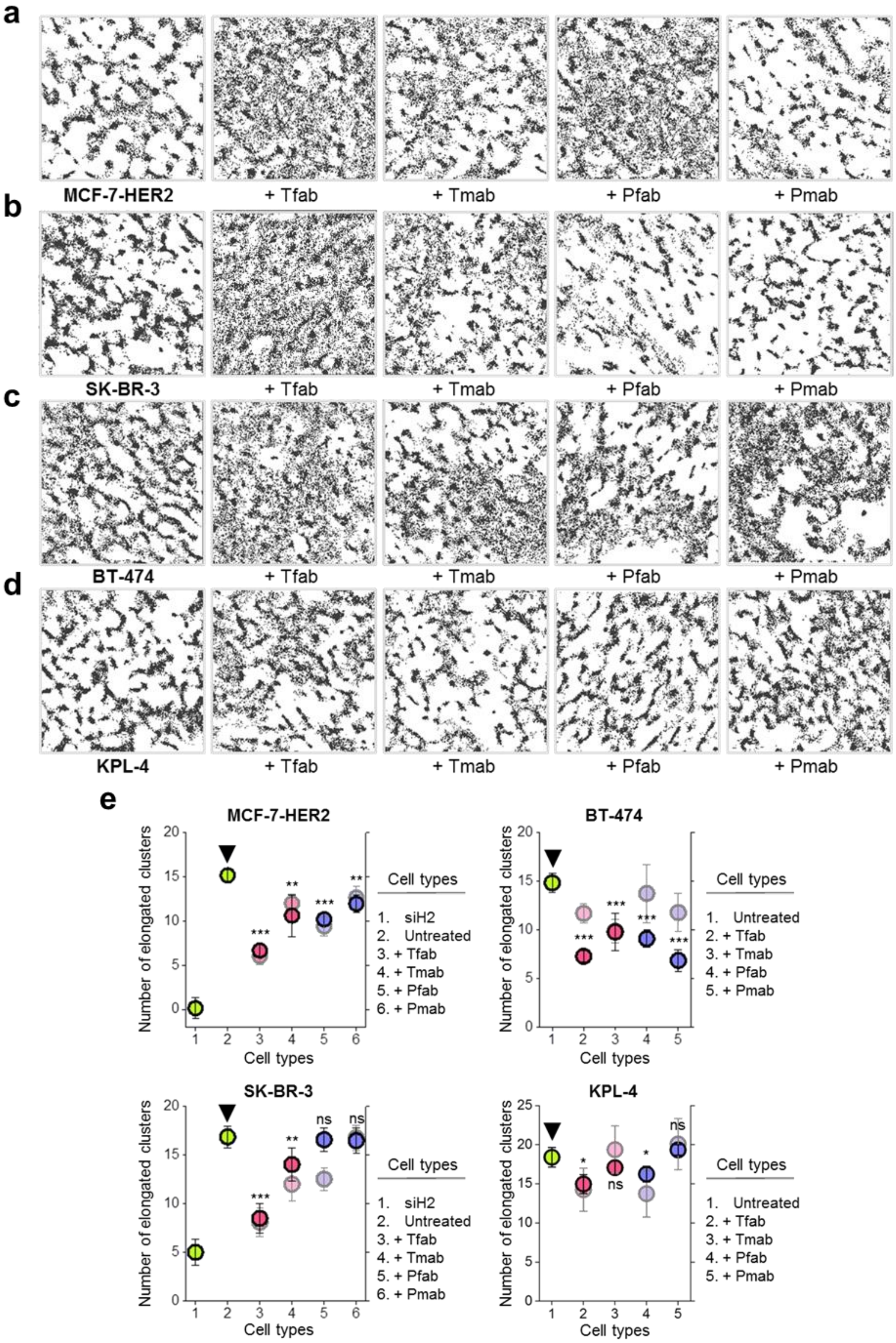

a
MCF-7-HER2
+ Tfab
+ Tmab
+ Pfab
+ Pmab
b
SK-BR-3
+ Tfab
+ Tmab
+ Pfab
+ Pmab
c
BT-474
+ Tfab
+ Tmab
+ Pfab
+ Pmab
d
KPL-4
+ Tfab
+ Tmab
+ Pfab
+ Pmab
e
MCF-7-HER2
Number of elongated clusters
Cell types
Cell types
1. siH2
2. Untreated
3. + Tfab
4. + Tmab
5. + Pfab
6. + Pmab
BT-474
Number of elongated clusters
Cell types
Cell types
1. Untreated
2. + Tfab
3. + Tmab
4. + Pfab
5. + Pmab
SK-BR-3
Number of elongated clusters
Cell types
Cell types
1. siH2
2. Untreated
3. + Tfab
4. + Tmab
5. + Pfab
6. + Pmab
KPL-4
Number of elongated clusters
Cell types
Cell types
1. Untreated
2. + Tfab
3. + Tmab
4. + Pfab
5. + Pmab

Supplementary Figure 5. HER2 antibodies can affect membrane deformation.

(a-d) Representative HER2 distribution patterns from 1 day treatments with trastuzumab fab (+ Tfab), trastuzumab (Tmab), pertuzumab fab (Pfab), and pertuzumab (Pmab), and untreated controls, on MCF-7-HER2 (a), SK-BR-3 (b), BT-474 (c), and KPL-4 (d) cells. (e) FLS densities in four high expressers without (green circles) and with 2 hr (light pink and violet circles) and 1 day (pink and violet circles) treatments of Tfab, Tmab, Pfab, and Pmab. The numbers of cells studied after 2 hr and 1 day after treatments are marked as *n* (2 hr) / *n* (1 day). We also used partial siRNA to reduce HER2 (siH2) from MCF-7-HER2 and SK-BR-3 cells to show the HER2 expression level dependent FLS densities. Inverted black triangles point to the parental high expressers. Similar distributions were found between the 2 hr and 1 day antibody/Fab treatment time points. In all high expressers, FLS densities were generally down-modulated by the four treatments, but the relative effects of individual treatments varied among different high expressers. KPL-4 was the least responsive. ***: P-value $< 10^{-6}$, **: P-value $< 10^{-4}$, and *: P-value $< 10^{-2}$ by two-sided, unpaired Welch's *t*-test. All values are mean ± s.e.m.

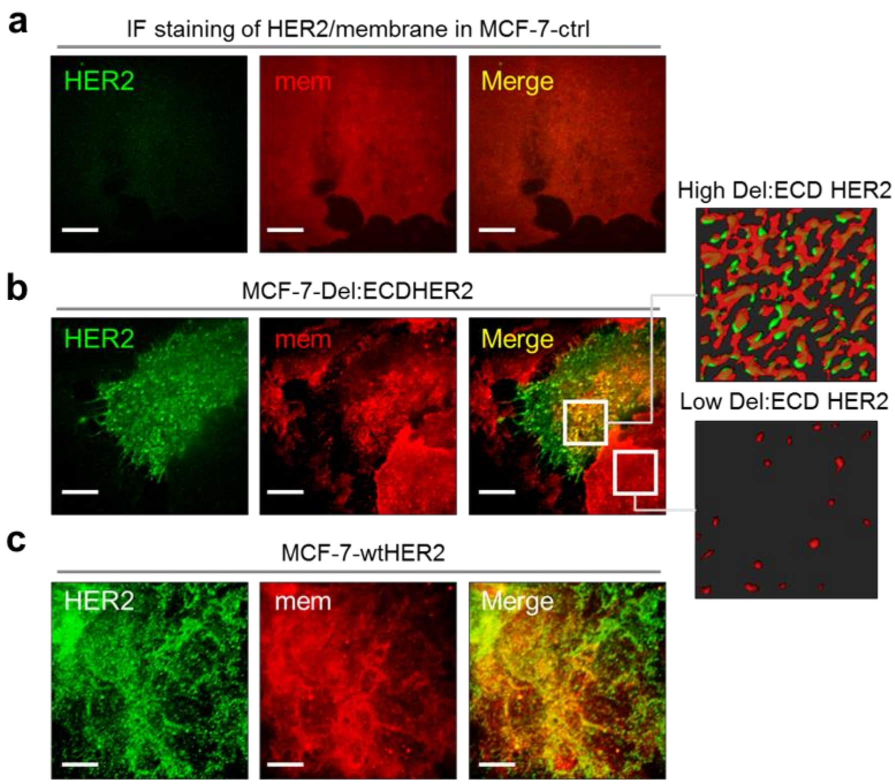

Supplementary Figure 6. Truncation of HER2 ECD does not significantly perturb membrane deformation

To investigate the effect of deletion of the HER2 ECD on membrane deformation, we compared the membranes of MCF-7 cells transiently transfected with an ECD deletion mutant (ΔECDHER2, b) with MCF-7-ctrl (MCF-7 transfected with the control vector, a) and MCF-7-wtHER2 (a stable cell line with wtHER2 overexpression, c). IF was performed using confocal microscopy to visualize the basal membranes and HER2 levels. The HER2 levels were assessed using an antibody that recognizes the HER2 c-terminal region (green). Membrane morphology was visualized using the same membrane dye as in Fig. 2c and Supplementary Fig. 2e. As expected, in MCF-7-ctrl (low HER2) the fluorescence intensity of the dye is essentially homogeneous throughout the cell surface (a), indicating that the membrane was largely flat. In

contrast, membranes of the transfectants with high ΔECDHER2 expression (b) appear very similar to those in MCF-7-wtHER2 cells (c). Both (high expressers of ΔECDHER2, and wtHER2) show non-uniform dye intensities, and these high intensity regions overlap well with regions containing optically concentrated HER2 (~ 80% overlap). In (b), the boxed areas show examples of cells with high (upper right) and low (lower right) expressions of ΔECDHER2 within the same field. These boxed areas were processed to clearly show the differences in the extent of membrane deformation between cells with high and low ΔECDHER2 expression.

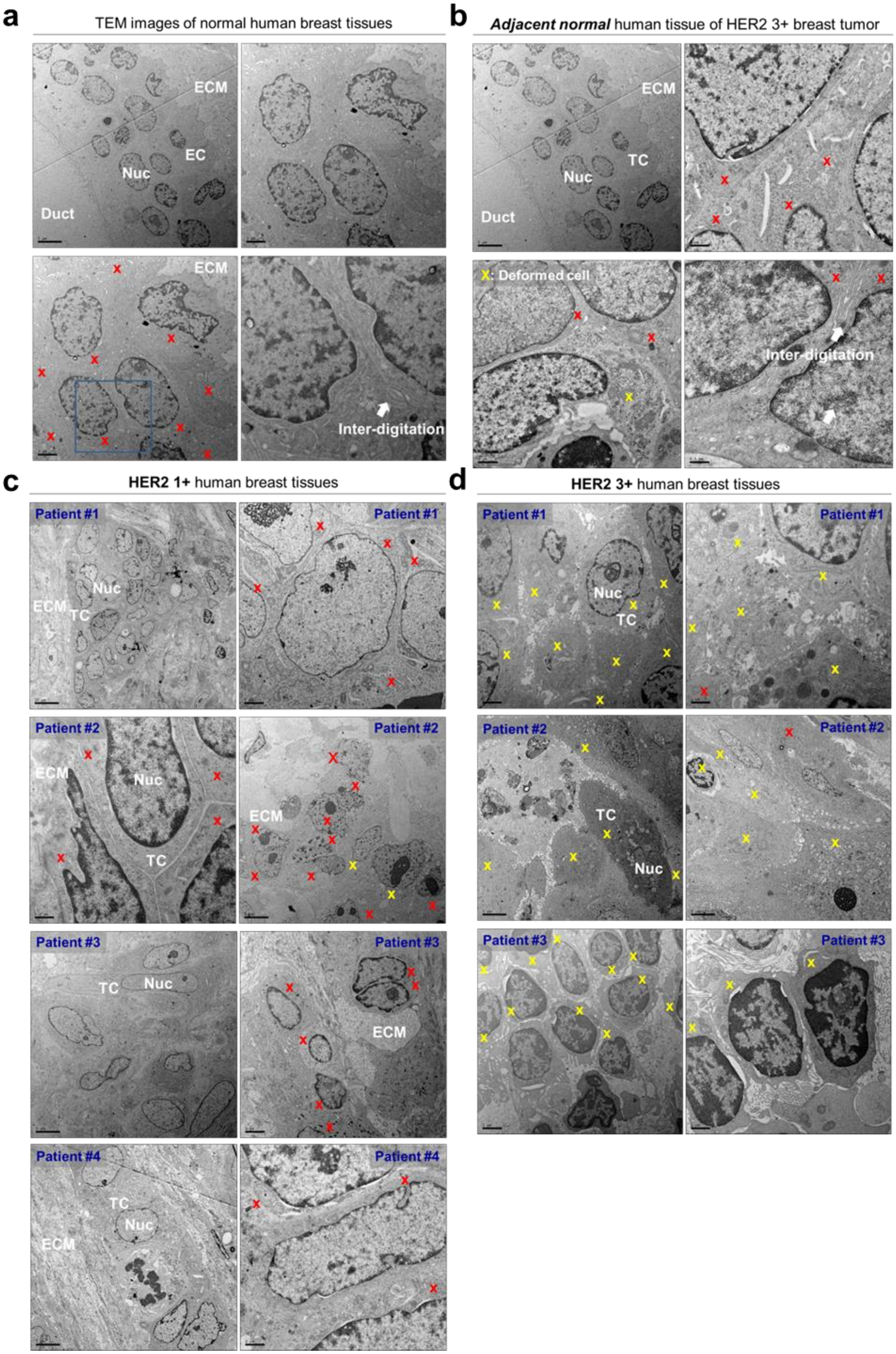
a
TEM images of normal human breast tissues
ECM
EC
Nuc
Duct
Inter-digitation
b
Adjacent normal human tissue of HER2 3+ breast tumor
ECM
TC
Nuc
Duct
X: Deformed cell
Inter-digitation
c
HER2 1+ human breast tissues
Patient #1
Patient #2
Patient #3
Patient #4
ECM
TC
Nuc
d
HER2 3+ human breast tissues
Patient #1
Patient #2
Patient #3
TC
Nuc

Supplementary Figure 7. HER2 3+ breast tumors show membrane deformation and disrupted epithelial polarity

We inspected morphologies of individual cells in different human breast tissue sections shown in panels (a) to (d). This inspection was performed independently and in a blinded manner by three individuals. Cells that had deformed cell membranes in any region and exhibited disrupted cell-cell interfaces were counted. Any cell that showed a nucleus and distinctive cell body was examined. The mean (± s.e.m.) ratio of deformed cells (marked with (yellow x)) versus the total number of cells (the sum of the deformed and normal cells (red x)) per each tissue type is shown in a plot in Fig. 4e. EC: epithelial cell, ECM: extracellular matrix, TC: tumor cell, and Nuc: nucleus. (a) Normal human breast tissue from a patient undergoing reduction mammoplasty. Epithelial polarity is maintained in these cells. All the cells in the upper right image are counted as normal, as indicated by the red x's in the duplicate lower left image. Some cells show finger-like structures that are inter-digitated (the right bottom image is an enlargement of the blue boxed area in the left bottom image) and maintain smooth cell-cell interfaces. (b) Adjacent normal human breast tissue from a HER2 3+ breast tumor (patient #2). Epithelial polarity is also well preserved. One cell is apoptotic (condensed nucleus) in the bottom left image and this cell was not counted. But the cell above the apoptotic cell shows disrupted membrane and perturbed cell interfaces. Some cells in the right bottom image show inter-digitated fingers and cell-cell interfaces are still smooth. (c) HER2 1+ breast ductal adenocarcinoma tissues of 4 patients. Most cells show well-maintained epithelial polarity similar to that in normal breast tissues, but occasionally a few cells show perturbed membranes and interfaces similar to what is seen in the cells of patient #2. (d) HER2 3+ breast ductal adenocarcinoma tissues of 3 patients. In all specimens, cell interfaces are visibly disrupted and exhibit deformed membranes with FLS.

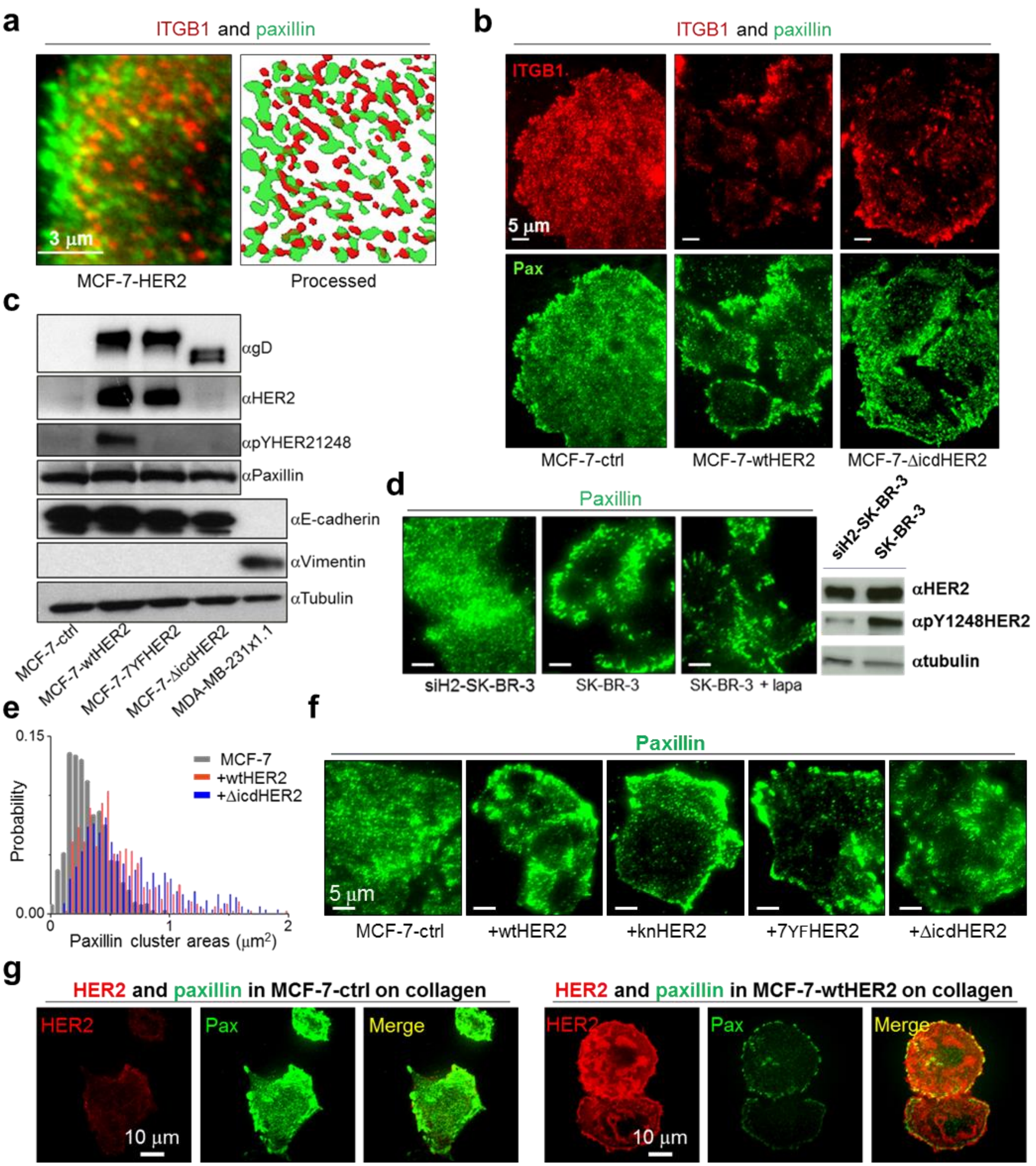
a
ITGB1 and paxillin
3 μm
MCF-7-HER2
Processed
b
ITGB1 and paxillin
ITGB1
5 μm
Pax
MCF-7-ctrl
MCF-7-wtHER2
MCF-7-ΔicdHER2
c
αgD
αHER2
αpYHER21248
αPaxillin
αE-cadherin
αVimentin
αTubulin
MCF-7-ctrl
MCF-7-wtHER2
MCF-7-7YFHER2
MCF-7-ΔicdHER2
MDA-MB-231x1.1
d
Paxillin
siH2-SK-BR-3
SK-BR-3
SK-BR-3 + lapa
siH2-SK-BR-3
SK-BR-3
αHER2
αpY1248HER2
αtubulin
e
0.15
0.00
Probability
MCF-7
+wtHER2
+ΔicdHER2
0
1
2
Paxillin cluster areas (μm2)
f
Paxillin
5 μm
MCF-7-ctrl
+wtHER2
+knHER2
+7YFHER2
+ΔicdHER2
g
HER2 and paxillin in MCF-7-ctrl on collagen
HER2
Pax
Merge
10 μm
HER2 and paxillin in MCF-7-wtHER2 on collagen
HER2
Pax
Merge
10 μm

Supplementary Figure 8. HER2 overexpression reduces cell surface coverage of paxillin independently of HER2 signaling.

(a) Paxillin clusters overlap but do not co-localize with elongated HER2 clusters. Immunofluorescence stainings of HER2 (red; αH2Fab:QD) and paxillin (green; αpaxillin:Alexa488) in MCF-7-HER2 cells imaged by TIRFM (left). Image processing, similar to what was used in Supplementary Fig. 2d was applied to these images, and the processed image (right) shows little overlap between paxillin and elongated HER2 clusters (~ 5% of the total areas are colocalized). (b) Reduced cell surface localization of integrin beta 1 and paxillin is associated with high HER2 expression, irrespective of HER2's signaling activities. IF stainings of integrin beta 1 (ITGB1) (red; αCD29:alexa568) and paxillin (green; αpaxillin:Alexa488) show that their cell surface localizations are lower in cells that express high levels of either wtHER2 (MCF-7-wtHER2, middle column), or a signaling incompetent mutant of HER2 (MCF-7-ΔicdHER2, right column), versus cells that express low levels of HER2 (MCF-7 cells transfected with an empty control vector (vctrl), left column). This was not a result of changes in the endogenous levels of paxillin, as shown by western blots of wtHER2, 7YFHER2, and ΔicdHER2, paxillin, E-cadherin, vimentin, and tubulin from MCF-7 transfectant lysates (c). The signaling-independent effect of HER2 overexpression does not down-regulate E-cadherin or paxillin in MCF-7-HER2 cells. The various forms of HER2 were detected with both an anti-HSV-gD antibody, which recognized the gD epitope tag fused to the N-termini of all forms of heterologously expressed HER2, and an antibody that recognized the C-terminus of HER2. A phospho-specific (pY1248) HER2 antibody was used to assess HER2 activation status. MDA-MB-231x1.1, a mesenchymal-like cell-line, was used as both a negative and positive control for E-cadherin and vimentin levels, respectively. Both E-cadherin and paxillin levels in the MCF-7

lysates were similar in all lanes and HER2 overexpression did not up-regulate vimentin expression. (d) Cell surface coverage of paxillin, assessed by IF imaging by TIRFM, was examined as a function of HER2 expression and signaling in SK-BR-3 cells. HER2 expression was reduced by partial siRNA knockdown of HER2 in SK-BR-3 cells (siH2-SK-BR-3) and HER2 signaling was lowered by 30 min treatment of lapatinib (+ lapa). The reduced expression along with the corresponding decrease in constitutive HER2 activity are shown by WB (right). Cells within the siH2-SK-BR-3 pool exhibiting HER2 expression levels comparable to those of MCF-7 (by labeling HER2) were chosen for monitoring by TIRFM. Paxillin much more densely covers the basal cell membranes in low expressers (left), than in high-expressers SK-BR-3 (middle) and SK-BR-3 + lapa (right). (e) Distributions of paxillin cluster areas in parental MCF-7 cells (gray bars), two high expresser transfectants, MCF-7-wtHER2 (red bars) and MCF-7-ΔicdHER2 (blue bars). In low expressers, small paxillin clusters densely covered central areas of the basal cell surfaces. In high HER2 expressers, irrespective of receptor signaling activities, densities of small paxillin clusters were reduced in the central regions and larger clusters appeared particularly in the cell peripheries (Fig. 5b). (f) IF images of paxillin distributions in basal cell membranes of MCF-7-vctrl (low expresser) and various high expressers that transiently overexpress wtHER2 and signaling-incompetent HER2 mutants. Paxillin distribution in MCF-7-vctrl is more uniform and dense than those in the high expressers regardless of HER2 signaling activities. (g) Comparison of paxillin distributions of MCF-7-ctrl and MCF-7-wtHER2 cells grown on collagen matrices. The same excitation setting was used to show the inverse relationship between HER2 (red) levels and paxillin (green) densities for these two cell lines.

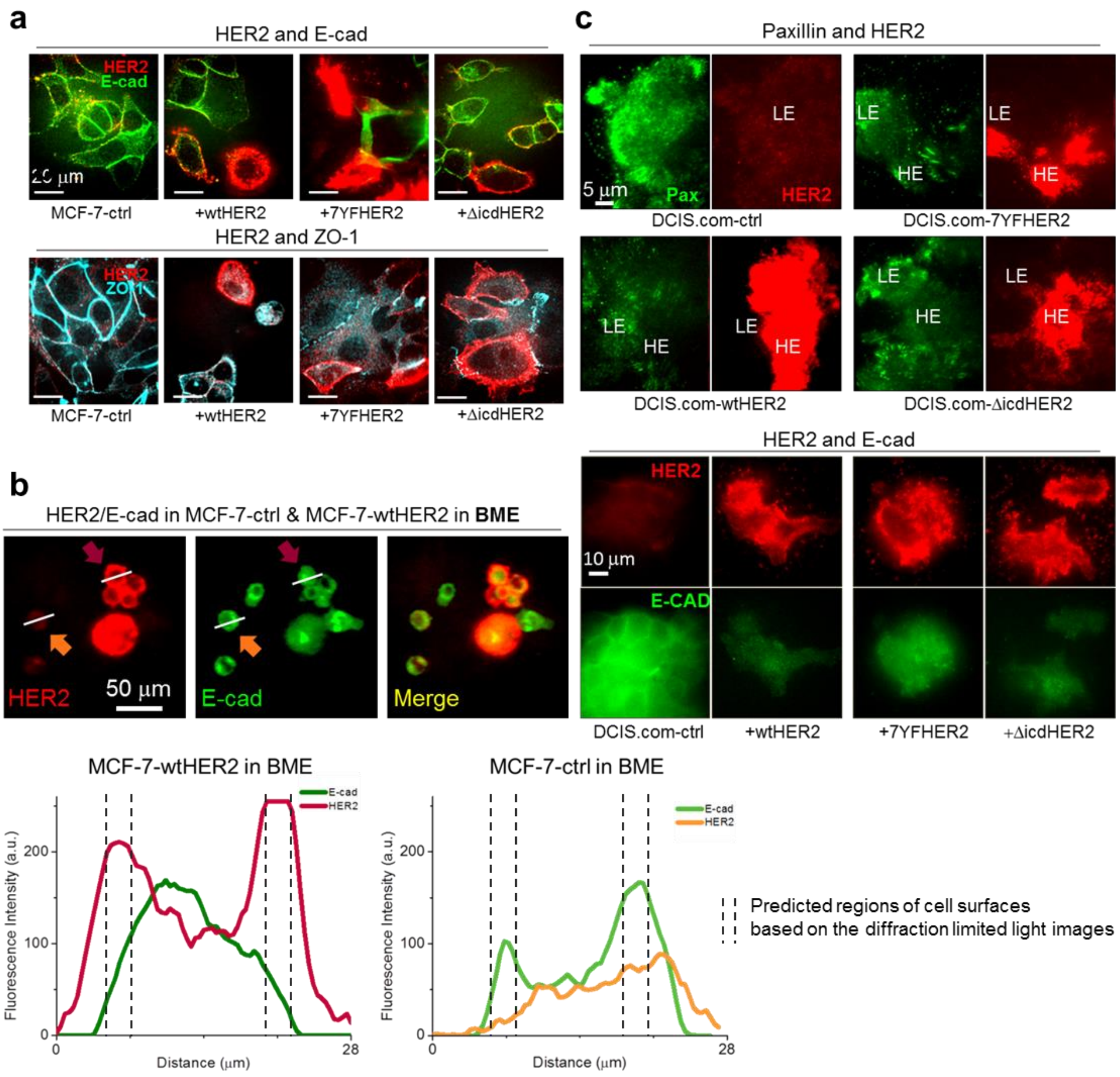

a
HER2 and E-cad
HER2
E-cad
MCF-7-ctrl
+wtHER2
+7YFHER2
+ΔicdHER2
HER2 and ZO-1
MCF-7-ctrl
+wtHER2
+7YFHER2
+ΔicdHER2
b
HER2/E-cad in MCF-7-ctrl & MCF-7-wtHER2 in BME
HER2
50 μm
E-cad
Merge
MCF-7-wtHER2 in BME
E-cad
HER2
Fluorescence Intensity (a.u.)
Distance (μm)
MCF-7-ctrl in BME
E-cad
HER2
Fluorescence Intensity (a.u.)
Distance (μm)
Predicted regions of cell surfaces
based on the diffraction limited light images
c
Paxillin and HER2
LE
HE
5 μm
Pax
HER2
DCIS.com-ctrl
DCIS.com-7YFHER2
DCIS.com-wtHER2
DCIS.com-ΔicdHER2
HER2 and E-cad
HER2
10 μm
E-CAD
DCIS.com-ctrl
+wtHER2
+7YFHER2
+ΔicdHER2

Supplementary Figure 9. HER2 overexpression disrupts cell-cell and cell-substrate interactions independently of HER2 signaling.

(a) Reduced cell surface localization of E-cadherin and ZO-1 in MCF-7 cells transiently expressing high levels of various forms of HER2. IF images (confocal imaging) of E-cadherin (green) or ZO-1 staining (cyan) in the wtHER2, 7YFHER2, and ΔicdHER2 transfectants, compared with a control vector transfectant. Relative HER2 expression levels are shown (red; αH2Fab:QD). E-cadherin and ZO-1 are clearly localized at the cell interfaces in MCF-7-ctrl, indicating solid cell-cell contacts. In contrast, these localizations are greatly reduced for all high HER2 expressers (red). (b) The surface localizations of HER2 (red) and E-cadherin (green) are inversely related. To visualize multiple cells in the same focal plane and in the thick BME layer on the cover glass, a confocal microscope with a 20x objective with numerical aperture (N.A.) = 0.45 and long working distance (w.d. = 7.4 mm) was used. MCF-7-ctrl and MCF-7-wtHER2 cells were co-cultured in BME to compare the surface HER2 level dependent E-cadherin localization. The localizations of these proteins are compared between MCF-7-ctrl (orange arrow) and MCF-7-wtHER2 (purple arrow) cells in the upper panels by showing the intensity profiles (indicated by the white lines) across the membranes in the lower panels. The graphs in the lower panels show that when HER2 levels (purple line) are high on the membranes, E-cadherin (green) is more cytosolic; and when the surface HER2 levels are low (orange line), E-cadherin (light green) is more localized on the cell membrane. (c) Reduced cell-substrate and cell-cell contacts with high HER2 expression in MCF10DCIS.com cell transfectants. IF imaging for paxillin (Top; green) by TIRFM, and E-cadherin (Bottom; green) by confocal microscopy, along with HER2 (red for both image sets) were performed on DCIS.com cells transfected with either a control vector (ctrl), wtHER2, 7YFHER2, or ΔicdHER2. Similar to the results seen with

MCF-7 cell transfectants in Supplementary Fig. 8b-f, reduced cell surface signals for paxillin and E-cadherin were observed for all high HER2 expressers regardless of their signaling status. This suggests that cell-substrate, and cell-cell contacts are also reduced in DCIS.com cells by HER2 overexpression via a signaling-independent morphological deformation of cell membranes. LE and HE: low and high expressers.
